# Neurosteroids: Structure-Uptake Relationships and Computational Modeling of Organic Anion Transporting Polypeptides (OATP)1A2

**DOI:** 10.3390/molecules26185662

**Published:** 2021-09-17

**Authors:** Santosh Kumar Adla, Arun Kumar Tonduru, Thales Kronenberger, Eva Kudova, Antti Poso, Kristiina M. Huttunen

**Affiliations:** 1 School of Pharmacy, Faculty of Health Sciences, University of Eastern Finland, P.O. Box 1627, 70211 Kuopio, Finland; arun.tonduru@uef.fi (A.K.T.); kronenberger7@gmail.com (T.K.); antti.poso@uef.fi (A.P.); kristiina.huttunen@uef.fi (K.M.H.); 2 Institute of Organic Chemistry and Biochemistry (IOCB), Czech Academy of Sciences, Flemingovo Namesti 542/2, 160 00 Prague, Czech Republic; eva.kudova@uochb.cas.cz; 3 Department of Medical Oncology and Pneumology, Internal Medicine VIII, University Hospital of Tübingen, Otfried-Müller-Strasse 14, 72076 Tübingen, Germany

**Keywords:** neurosteroid, cellular uptake, Organic Anion Transporting Polypeptides (OATPs), docking, molecular modeling

## Abstract

In this study, we investigated the delivery of synthetic neurosteroids into MCF-7 human breast adenocarcinoma cells via Organic Anionic Transporting Polypeptides (OATPs) (pH 7.4 and 5.5) to identify the structural components required for OATP-mediated cellular uptake and to get insight into brain drug delivery. Then, we identified structure-uptake relationships using in-house developed OATP1A2 homology model to predict binding sites and modes for the ligands. These binding modes were studied by molecular dynamics simulations to rationalize the experimental results. Our results show that carboxylic acid needs to be at least at 3 carbon-carbon bonds distance from amide bond at the C-3 position of the androstane skeleton and have an amino group to avoid efflux transport. Replacement of hydroxyl group at C-3 with any of the 3, 4, and 5-carbon chained terminal carboxylic groups improved the affinity. We attribute this to polar interactions between carboxylic acid and side-chains of Lys33 and Arg556. The additional amine group showed interactions with Glu172 and Glu200. Based on transporter capacities and efficacies, it could be speculated that the functionalization of acetyl group at the C-17 position of the steroidal skeleton might be explored further to enable OAT1A2-mediated delivery of neurosteroids into the cells and also across the blood-brain barrier.

## 1. Introduction

Alzheimer’s disease (AD) is the most complex neurodegenerative disorder, leading to gradual loss of cognition and eventually death. In AD-brain, beta-amyloid (Aβ) deposits accumulate into neurons, making neurons lose their capacity to respond to stimuli and become proinflammatory contributing to neuronal death. So far, available drugs (donepezil [1,2] rivastigmine [3,4] galantamine [5,6], and memantine [7,8]) can partially treat only some symptoms of AD. Despite considerable research, currently, there is no cure for AD. Improper drug delivery combined with unwanted peripheral side effects causes late-stage clinical trial failures, and almost all clinical trials for AD-modifying drugs have failed. Central nervous system (CNS) diseases often meet the limited penetration of drugs by the blood-brain-barrier (BBB) [9]. Over 98% of drugs do not cross BBB at acceptable concentrations for therapeutic treatment. Thus, the BBB protects the brain tissue from xenobiotics and microbes, while allowing movement of oxygen and essential substances via specific transporters [10]. Carrier-mediated active uptake is also the primary transport mechanism for most of the CNS-drugs [11], and endogenous solute carriers (SLCs) have a major role in their CNS disposition [12,13].

For transporter-mediated drug delivery, drugs must have necessary structural features to serve as a selective substrate for the target transporter. Increasing the lipophilic nature of a molecule is not necessarily a workable alternative to improve the cellular or brain permeability of CNS-drugs [14]. Instead, for carrier-mediated drug delivery, drugs must possess the crucial structural components to serve as a selective substrate for the target transporter protein. To use influx transporters purely, these features need to be recognized and incorporated in molecular design during the early steps of drug development, while trying to avoid the features that may turn the molecule into an efflux substrate [15].

Organic Anionic Transporting Polypeptides (OATPs) are transporter proteins that mediate CNS-delivery of endogenous compounds. In particular, OATPs can transport relatively large substrates, such as thyroid (T_3_ and T_4_) and steroid hormones, and are more suitable carriers for larger drugs, such as estrone-3-sulfate, estradiol-17β-glucuronide and pregnanolone sulfate [15]. Although different subtypes of OATPs are broadly distributed throughout the body, specific isoforms, OATP1A2, OATP2B1, OATP1C1, and OATP3A1, are highly expressed in the brain [14]. Some of OATPs may also have several substrate binding sites, which increase the possibilities for efficient and selective drug release [14].

Neurosteroids are de novo synthesized in the brain and exhibit potent neuromodulatory physiological functions, such as memory development, cognition, and neuroprotection [16,17,18]. The behavioral and systemic action of neurosteroids can be attributed to their direct modulatory effects on neurotransmitter receptors, such as the GABA_A_ (γ-aminobutyric acid-A) [19], glycine [20], AMPA (α-amino-3-hydroxy-5-methyl-4-isoxazolepropionic acid) [21], and NMDA (*N*-Methyl-*d*-aspartic acid) receptors [20,22]. Prolonged activation of these receptors can lead to cell death (excitotoxicity), and their abnormal regulation may cause neuropathological conditions as seen, for example, in AD, ischemia, or traumatic brain injury [23,24,25]. Activity of these receptors can be influenced by several allosteric steroidal modulators synthesized in the nervous tissue from cholesterol, or steroidal precursors from peripheral sources [26,27].

However, it has been long known that not all steroids are delivered efficiently into the brain via OATPs; there are enormous variations and selectivity on brain uptake of steroid hormones, which has been directly linked to the structural features of these compounds [10]. Moreover, P-glycoprotein (P-gp) from ATP-Binding Cassette (ABC) membrane-associated transporters and organic anion transporter 3 (OAT3) from SLCs actively efflux steroids at the BBB [28,29,30]. The brain bioavailability of administered steroids is also poor because of their metabolic instability [31]. If the structural features required for brain-selective OATP-mediated uptake that avoids major efflux transport will be recognized, the delivery of novel neuroprotective steroids across the BBB could be increased. However, lack of robust computational approaches to fine-tune the rational drug design on selective delivery of synthetic neurosteroids remains a challenge for early drug discovery.

**Figure 1:**
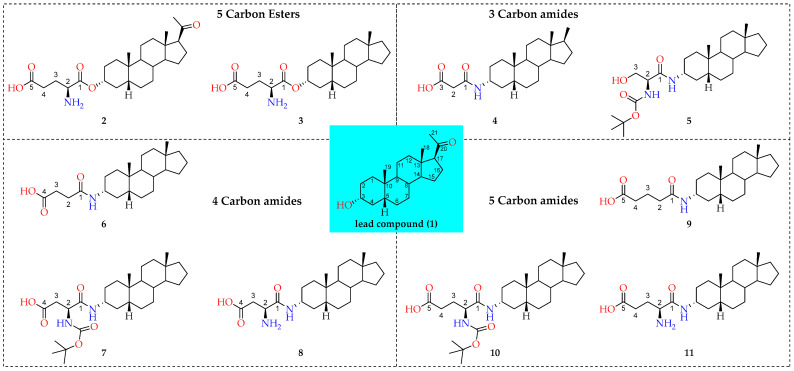
Structures of studied compounds.

In this study, we investigated (1) the efficient delivery of synthetic neurosteroids **1–11** (Figure 1) via OATPs to identify the structural components required for OATP-mediated cellular uptake, (2) identifying structure-uptake relationships for them to rationalize the experimental results and hypotheses with rigorous computational approaches. Computational methods were employed to investigate the binding of synthetic neurosteroids using in-house developed homology models to identify the putative binding site within OATP1A2 and molecular dynamics simulations to study the stability of their binding. OATP1A2 was chosen as a relevant model to study the OATP transport mechanism, due to the comprehensive biochemical/affinity data available. These investigations help our understanding of binding patterns and probable mechanism of transport of neurosteroids via OATP1A2.

## 2. Results

### 2.1. Cellular Uptake Studies

Cellular uptake of synthetic neurosteroids **1**–**11** (syntheses have been reported in our earlier publications [32,33]) was evaluated by using the MCF-7 (human breast adenocarcinoma; Michigan Cancer Foundation-7) cell line, which is known to express several OATPs and efflux transporters [34,35]. More detailed transport mechanisms were investigated by performing Eadie-Hofstee plot analysis, which revealed that almost all synthesized neurosteroids **1–11** had two distinct transport mechanisms.

#### 2.1.1. Cellular Uptake of 3α5β-Pregnanolone (**1**, CAS Number 128-20-1)

**Figure 2:**
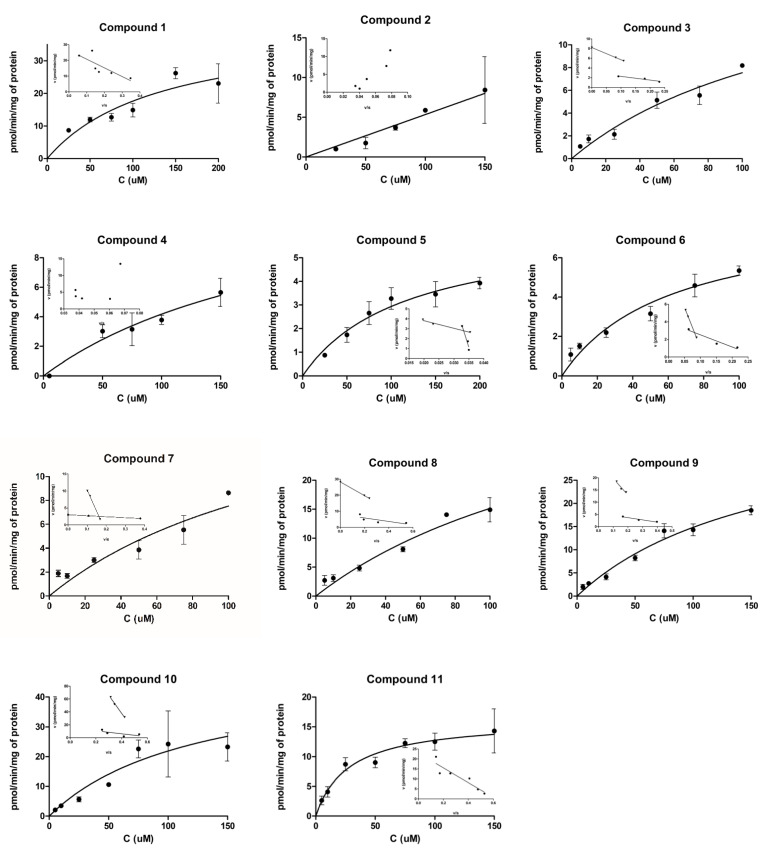
Cellular uptake of compounds **1**–**11** into the MCF-7 cells over a concentration range of 5–200 µM and the Eadie-Hofstee plots for transporter-mediated uptake (insets). The data is presented as mean ±SD (*n* = 3).

Structurally, 3α5β-Pregnanolone (**1**) has a α-hydroxy group at the C-3 position, the configuration of the proton at the C-5 position is beta, and it has an acetyl group at the C-17 position of the androstane skeleton. 3α5β-Pregnanolone (**1**) was transported with high efficacy into the cells (V_max_ 26.3 ± 4.2 pmol/mg/min; Figure 2, Table 1).

#### 2.1.2. Cellular Uptake of Neurosteroids with Ester Bond (**2** and **3**)

Compound **2** is pregnane skeleton, while compound **3** is androstane. In both instances of **2** and **3**, the 3α-hydroxy group was transformed into 5-carbon containing 3α-glutamate ester moiety, while the acetyl group at the C-17 position was absent in the case of **3**.

For compound **2**, because of the non-linearity of the plots, we could not calculate pharmacokinetics parameters from Eadie-Hofstee analysis. According to Figure 2, the compound did not reach the saturation stage, and, together with Eadie-Hofstee analysis, this may imply that this compound can induce the transporter’s function or expression [36]. Another explanation is that the two broadly overlapping transport mechanisms cannot be separated from the Eadie-Hofstee plots. According to the cellular uptake results, compound **3** used two transport mechanisms, but, interestingly, these mechanisms were equally efficient (V_max_/K_m_ values 0.39 and 0.33), implying to two different OATP transporter subtypes, rather than two distinct binding sites. However, both mechanisms also had relatively low capacities (V_max_ 3.0 and 8.2 pmol/mg/min), and compound **3** had three-times greater affinity for the lower capacity transporter (25.2 vs. 7.7 µM; Figure 2, Table 1).

#### 2.1.3. Cellular Uptake of Neurosteroids Having 3-Carbon Amide Residues (**4** and **5**)

In this series of compounds, the ester linkage was replaced by an amide. Besides the regular 3α5β-configuration, the amide linker was a short 3-carbon residue. Compound **4** had a methyl group at the C-17 position of the androstane skeleton, malonamide residue at the C-3 position, and a terminal free carboxylic acid at a distance of 3 carbons from the amide bond. In the case of compound **5**, the androstane skeleton was functionalized with a *l*-serinamide moiety at the C-3 position, while the amino group of the *L*-serinamide was protected by a *tert*-butyloxycarbonyl (Boc-) group.

Similar to compound **2**, compound **4** had almost a linear uptake profile (Figure 2). Therefore, the affinity of **4** (K_m_ value ca. 233 µM) cannot be accurately reported. Contrarily, compound **5** was transported with a low capacity and efficiency mechanism (V_max_ 5.3 pmol/mg/min, K_m_ 76.4 µM, and V_max_/K_m_ value of 0.07). According to the Eadie-Hofstee analysis, compound **5** was also able to use another very low affinity mechanism with higher capacity (Figure 2, Table 1), which may indicate that this compound had two binding sites in the same transporter. However, it is unlikely that the latter transport mechanism would be significant with therapeutically relevant concentration (K_m_ value ca. 960 µM). Compared to all other studied synthetic neurosteroids, the C-17-methyl group in compound **4** was noticed to influence the uptake efficiency.

#### 2.1.4. Cellular Uptake of Neurosteroids Having Medium Length 4-Carbon Amide Residues (**6**, **7**, and **8**)

Structurally, in this next batch of compounds, the androstane core of steroids were unchanged from 3α5β-configuration, and all the compounds possessed a medium length 4-carbon amide residue at the C-3 position of the steroidal skeleton. Compound **6** had a hemisuccinate moiety connected via amide bond at C-3, compound **7** had a Boc-protected-aspartamide, and compound **8** contained an *L*-aspartamide group.

Compound **6** had a low capacity but very efficient transport mechanism because of its high affinity for this mechanism (V_max_ 3.0 pmol/mg/min, K_m_ 3.0 µM, and V_max_/K_m_ value of 1.0; Figure 2, Table 1). Furthermore, another transport mechanism (greater capacity but lower affinity, most likely another binding site in same transporter) could carry this compound into the cells but with relatively low efficiency (V_max_/K_m_ value of 0.18). Compound **7** was also transported by two different mechanisms, similar to compound **6**. However, both transport mechanisms were relatively inefficient (V_max_ 3.9 and 10.2 pmol/mg/min and V_max_/K_m_ values 0.28 and 0.11, respectively; Figure 2, Table 1). Similar to compound **3**, compound **8** had also two transport mechanisms (or OATP-subtypes involved) that were almost equally efficient (V_max_/K_m_ values 0.73 and 0.66). These transport mechanisms were relatively efficient, and twice as efficient as compound **3** (V_max_ 8.9 and 28.2 pmol/mg/min; Figure 2, Table 1).

#### 2.1.5. Cellular Uptake of Neurosteroids with Longer Length 5-Carbon Amide Residues (**9**, **10**, and **11**)

Finally, the C-3 position of the 3α5β-androstane skeleton structure was further homologated to long length 5-carbon amide residues. Compound **9** contained a hemiglutarate moiety attached via amide bond at C-3. Compound **10** had a Boc-protected-L-glutamate, while compound **11** possessed a *L*-glutamate moiety attached via amide bond.

Similar to compound **5**, compound **9** could use two transport mechanisms (two binding sites in same transporter), from which only the other was relevant. This transport mechanism had low capacity but reasonable efficiency (V_max_ 5.5 pmol/mg/min and V_max_/K_m_ value of 0.60, respectively; Figure 2, Table 1). Compound **10** had also two transport mechanisms (two binding sites) with different capacities and efficiencies, similar to compounds **6** and **7** (V_max_ 5.5 and 26.5 pmol/mg/min and V_max_/K_m_ values 0.60 and 0.39, respectively; Figure 2, Table 1). Besides 3α5β-pregnanolone, only one other compound (**11**) was transported by a single transport mechanism V_max_ 17.2 ± 2.1 pmol/mg/min; Figure 2, Table 1). Because of the higher affinity for its primary transporter (K_m_ value 24.2 µM compared to the one of compound **1**; 55.4 µM), compound **11** had relatively high transport efficiency (V_max_/K_m_ value of 0.71 compared to compound **1**; 0.47).

#### 2.1.6. Transporter-Mediated Uptake of Neurosteroids **1**–**11** at pH 5.5

**Figure 3:**
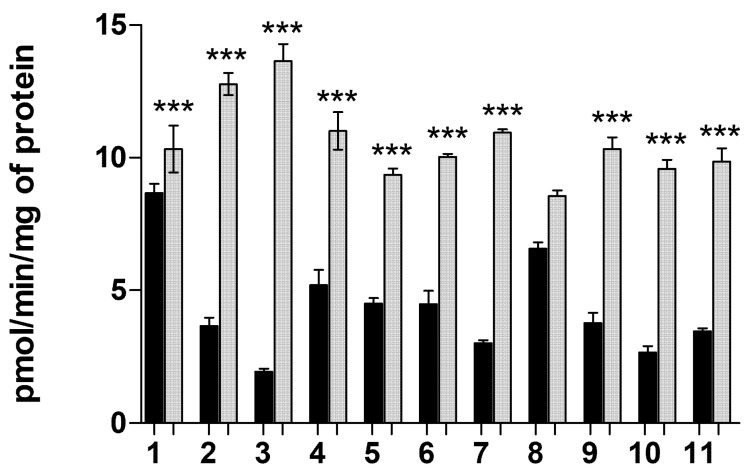
Cellular uptake of 25 µM compounds **1**–**11** into the MCF-7 cells at pH 7.4 (black bars) and at pH 5.5 (white bars). The data is presented as mean ± SD, *n* = 3 (*** *p* < 0.001, one-way ANOVA, followed by Tukey’s test).

Transporter-mediated uptake was also evaluated at pH 5.5, which is optimal for OATP-mediated transport [35]. These uptakes were compared to the uptake at pH 7.4 (Figure 3). It can be concluded that the decreased pH increased the uptake of all neurosteroids, except of compound **8**. This shows that OATPs were mainly responsible for the transport of these neurosteroids into the cells.

#### 2.1.7. Transporter-Mediated Uptake of Neurosteroids **1**–**11** at pH 7.4

**Figure 4:**
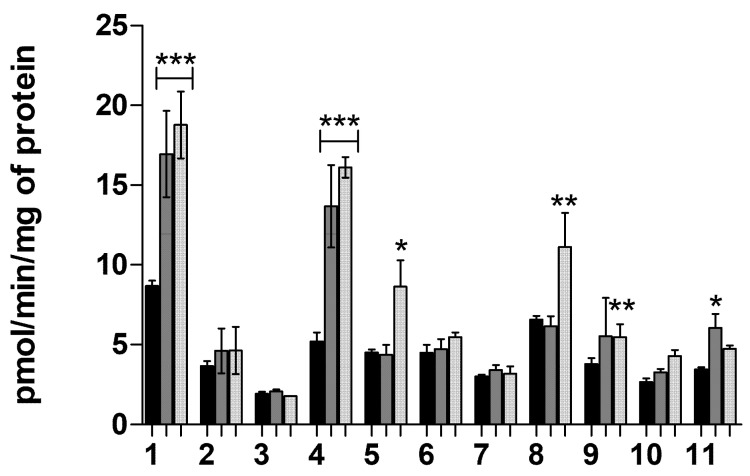
Cellular uptake of 25 µM compounds **1**–**11** into the MCF-7 cells at pH 7.4 in the absence (black bars), and presence of efflux inhibitors (50 µM), elacridar (P-gp and BCRP inhibitor, white bars), and MK-571 (unselective MRP inhibitor, dotted bars). The data is presented as mean ± SD, *n* = 3 (* *p* < 0.05, ** *p* < 0.01, *** *p* < 0.001, one-way ANOVA, followed by Tukey’s test).

Cellular uptake of neurosteroids (25 µM) was further studied at pH 7.4 when the cells were pre-incubated with known efflux inhibitors, elacridar (P-glycoprotein (P-gp) and breast cancer resistant protein, (BCRP)), and MK-571 (multidrug resistance proteins, MRPs) to investigate if the compounds are substrates to these efflux transporters. The uptake of compounds **2**, **3**, **6**, **7**, **10** was not affected either by elacridar or MK-571, whereas the uptake of compounds **1** (3α5β-pregnanolone), **4**, **5**, **8**, **9**, and **11** was increased significantly in the presence of these efflux inhibitors (Figure 4).

### 2.2. Molecular Modeling

#### 2.2.1. Homology Model—OATP1A2 Model Shows Two Binding Sites

**Figure 5:**
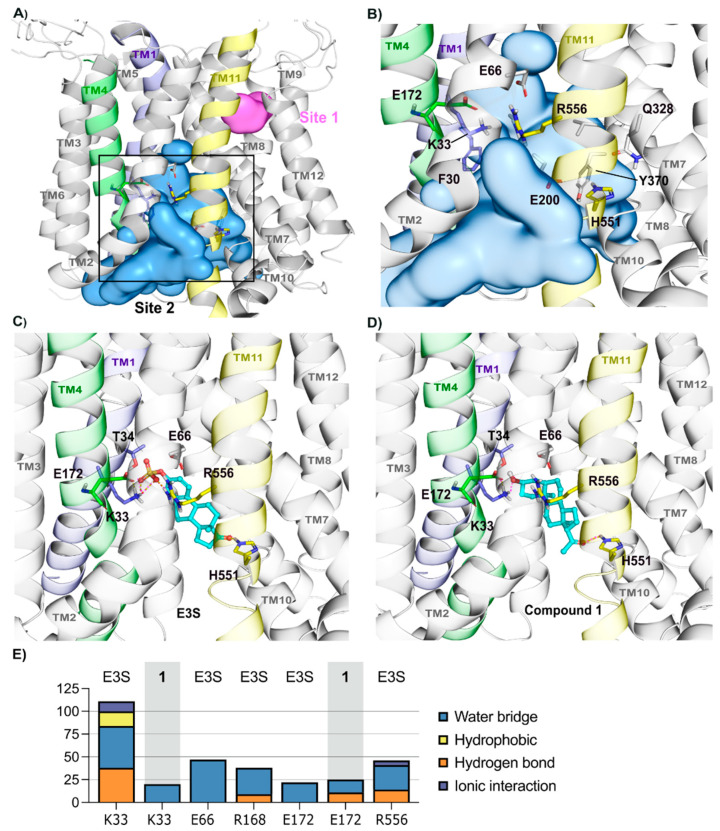
Human OATP1A2 homology model and its conserved potential binding pocket. Overview of the OATP1A2 model highlighting the volume of the two predicted binding pockets (Site 1, in pink, and Site 2, in blue) (**A**) followed by an insight of Site 2 (**B**). Predictions were performed using SiteMap and pockets were selected according to their druggability score, as described in the methods section. The compounds E3S and 1 were docked and simulated within the Site 2 and representative frames are depicted in (**C**) E3S and (**D**) compound 1, where one can observe the main residues with stable interactions in the simulations lying in the transmembrane helices 1 (highlighted in light purple), 4 (in green), and 11 (in yellow). (**E**) Individual residues and their interaction frequencies (% of simulation time) for each compound are shown as bar plots, where each interaction type is colored differently.

OATP1A2 transporter model structure was validated and deposited in the Zenodo repository. Potential binding sites within the transporter open cavity were predicted using SiteMap, suggesting a large binding site (see Supporting information, Table S1), namely Site 1 and 2 (Figure 5A). Site 2 is in the central pore region involved by all the transmembrane helices (TH), with exception of TH3, TH6, and TH9 (Figure 5A,B). This site also contains relevant residues for the substrate transport of OATP1A2, such as E172 [37], R168 [38], and other highly conserved residues, such as Arg556 (conserved in all OATPs; see Supporting information, Figure S3B) and Lys33 (within OATP1 family, Figure 5B; also see Supporting information, Figure S3A). These positively charged residues, corresponding to Arg580 and Lys41 in OATP1B1 [39] and OATP1B3 [40], play a role in the uptake of substrates. Given the relevance of the predicted pocket, the potential binding mode of synthetic compounds (**1–11**) was suggested by docking along with the reference substrate Estrone-3-Sulfate (E3S) [38].

#### 2.2.2. OATP1A2 Substrate E3S Displays Two Potential Binding Modes

The reference substrate E3S (efficacy = 10.21, K_m_ = 6.5 µM, V_max_ = 66 ± 8.5 [39]) was evaluated as a model substrate for the steroidal derivatives, using docking and a total of 4 µs MD simulations. E3S docking pose was selected based on its interactions with previously described relevant residues for the binding of this substrate [39,40,41,42,43], namely Lys33, Arg556, Arg168, and Glu172. E3S initial binding mode (Figure 5C,E) displays its sulfate group oriented toward the extracellular region, interacting with Lys33, Arg556, and its carbonyl group oriented toward the intracellular side of the pocket, interacting with His551.

E3S displayed two potential binding modes during the 4 µs MD simulation. Initial pose with the sulfate group toward the extracellular side was observed for around 75% of time, and the second pose with the sulfate group facing toward the intracellular side approximately 25% of the time. During the MD simulation, E3S interaction with Lys33 is basically constant (100% of simulation time). This is in line with reports that Lys33 is crucial for the uptake of substrates in OATP1B1 [39] and OATP1B3 [40]. Glu66 and Arg556 residues also display polar interactions for >50% of the simulation time (Figure 5E). Lys33 and Arg556 formed hydrogen bonding with the sulfate group of the substrate, and Glu66 formed water bridges with the sulfate group. E3S also formed hydrophobic interactions with residues, such as Arg168, Phe332, and Phe336. As this initial analysis supports our homology and binding site model for E3S, and since this result was in line with models reported in the literature [40], synthetic neurosteroid compounds (**1**–**11**) were docked into the model to predict the binding of these ligands into OATP1B1 [37] and OATP1B3 [40]

The 3α5β-pregnanolone (**1**) exhibited a similar binding mode to E3S in docking (Figure 5D,E). However, it does not show stable interactions during the 2-µs simulation with conserved residues Lys33 and Arg556 and displayed interactions with Ser525 and Phe532 (50% of the simulation time). The unstable binding pose reconciles the compound’s low affinity (K_m_ = 55.4 µM).

#### 2.2.3. Binding of Compounds **2** and **3**

The glutamate moiety of compounds **2** and **3** acts as bioisostere for the sulfate group in E3S. Compounds **2** and **3** showed similar binding poses and interactions during the docking with OATP1A2 model. The simulation data revealed an additional interaction in compound **3** carboxylic group with Arg168. Further, compound **2** distorted the helix structures of TH8 because of the water bridge. The docking poses are shown in Supporting information, Figure S4A,B,E.

#### 2.2.4. Binding of Compounds **4** and **5**

Compound **4** showed hydrogen bond interactions with the Lys33 and Arg556 for 100% of the total simulation time (1.5 µs), whereas compound **5** showed water bridges with residues Lys33 and Glu66 during 40% of the simulation time. This compound also showed H-bond interaction with Leu526 and Asn41. The docking poses are shown in Supporting information, Figure S4C,D,F.

#### 2.2.5. Binding of Compounds **6**, **7**, and **8**

**Figure 6:**
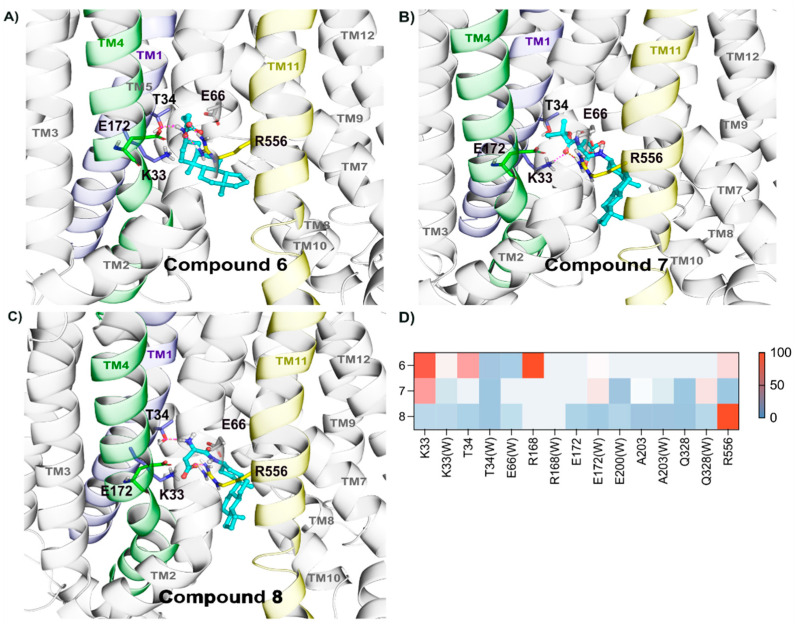
Stable binding mode of compounds **6**–**8** relies on polar contacts with Lys33, Arg168, and Arg556. Compounds **6** (**A**), **7** (**B**), and **8** (**C**) were docked and simulated within Site 2 of OATP1A2, and representative frames are depicted in (**A**–**C**), where one can observe the main residues with stable interactions in the simulations lying in the transmembrane helices 1 (highlighted in light purple), 4 (in green), and 11 (in yellow). (**D**) Individual residues and hydrogen bond interactions, and water interactions (represented as W), for each compound, are shown as heatmaps, where each interaction is colored according to the frequency.

Compounds **6**, **7**, and **8** displayed hydrogen bond interactions with Lys33 for 90%, 80%, and 40% of the simulation times, respectively (Figure 6A–D). Although side chain carboxylic acid moiety of compounds **6** formed hydrogen bonds with Arg168, Arg556, and Thr34 (around 80% of the simulation time), the efficiency was low (V_max_/K_m_: 0.28). This might be because of the conformational strain of the side chain in the binding region.

Interestingly, compound **7** was stabilized in a single conformation by polar contacts with Lys33, Ala203, Gln328, and Arg556 (Figure 6B). Among all the compounds (**1**–**11**), only compound **7** showed interactions with Ala203 (50% of the simulation time). Additionally, compound **7** also formed water bridges with conserved residue Glu172, which might have helped in its having better efficacy than other compounds (V_max_/K_m_ 1.0). Compound **8** was also stable in the binding region by interacting with the Arg556 residue (>100%). Nonetheless, other conserved residues interactions were not prominent.

#### 2.2.6. Binding of Compounds **9**, **10**, and **11**

**Figure 7:**
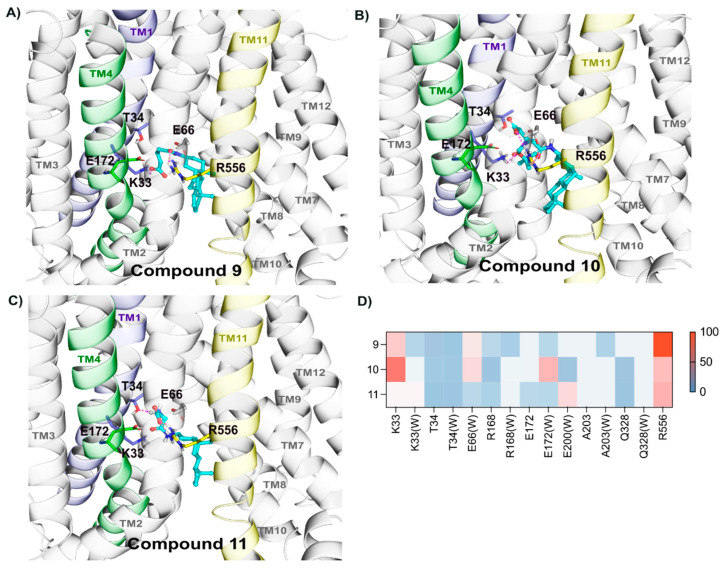
Stable binding mode of compounds **9**–**11** relies on polar contacts with Lys33 and Arg556. Compounds **9** (**A**), **10** (**B**), and **11** (**C**) were docked and simulated within the Site 2 of OATP1A2, and representative frames are depicted in (**A**–**C**), where one can observe the main residues with stable interactions in the simulations lying in the transmembrane helices 1 (highlighted in light purple), 4 (in green), and 11 (in yellow). (**D**) Individual residues and hydrogen bond interactions, and water interactions (represented as W), for each compound, are shown as heatmaps, where each interaction is colored according to the frequency.

Although these compounds exhibited similar efficacy compared to compounds **6**, **7**, and **8**, the transport was high for compound **11** (V_max_ 17.2 ± 2.1) and **10** (V_max_ 15.4 ± 6.5). Compound **11** also showed a similar interaction pattern by forming hydrogen bonds with Arg556 (70%), Lys33 (50), and Glu172 (30%) (Figure 7A–D). Likewise, compound **9** and **10** also displayed interactions with Arg556 and Lys33 for the highest frequency of the simulation time (Figure 7A,B,D). Furthermore, compound **10** also formed interactions with Threonine residues 34, 552, and 555, while compound **9** with Arg168.

#### 2.2.7. Principal Component Analysis (PCA)

Principal component analysis was performed to observe the conformational flexibility of the protein from overall molecular dynamics simulation. The first two PCs, PC1 (21.83%) and PC2 (17.81%), contributed to explaining up to 40% of the trajectory variance in the protein motions. The principal components (PC) generated can be represented as two sets of extreme motions of the protein, which were visually analyzed.

PC1 displays the most relevant protein motions, where the transmembrane helices tend to close towards each other separating the simulation trajectory into two states, herein named as open- and closed-states. An exception to this motion is the TM8, which moves in the opposite direction than the other transmembrane helices. Inspired by this observation, we investigated the distances between the helices’ center of mass and compared them among the simulations with different ligands. We observed that both E3S and compound 11 behaved similarly in most of the cases, i.e., they have the open conformation (one quartile of distances shows open conformation) compared to others. The distance between TM10 and TM8 for E3S and compound 10 suggests a more open conformation of OATP1A2, while compound 1 would stabilize the closed conformation.

## 3. Discussion

Compounds **1** and **4** were substrates for all studied efflux transporters, showing that derivatization to C-17 carbon may increase the affinity for efflux transporters and does not have any major effect on the OATP-mediated uptake. However, with appropriate residue at C-3 carbon, the efflux phenomenon can be abolished, which was the case with **2**. Compounds **5** and **8** were substrates only to the MRP family, but not to elacridar-sensitive efflux transporters, while compound **11** was elacridar-sensitive. Although the increase in the uptake was not significant because of the high variation, compound **9** had affinity at least to MRPs, most likely also to elacridar-sensitive transporters.

**Figure 8:**
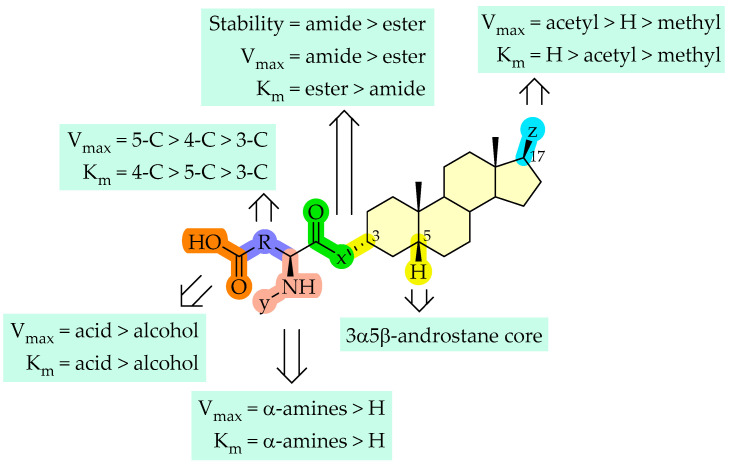
Summary of structure-uptake relationships of neurosteroids.

Overall, carboxylic acids need to be at least at 3 carbon-carbon bonds distance from amide bond at the C-3 position of the androstane skeleton and have an amino group (either free or protected) to avoid efflux transport (**3** and **10** versus **9** and **11**). In addition, the ester linkage may have benefits to avoid efflux transporter (**3** versus **11**), albeit it increases the instability of the compounds. If the distance of the carboxylic acid is only 2 carbon-carbon bonds, then the amino group should not be present, or it should be shielded with another groups, as with **6** and **7** versus **8**. In the absence of α-amino group on the residual C-3 substituent, the number of methylene bridges linked to the terminal carboxylic acids does not show much effect on transport capacity as in case of **6** and **9**. Boc-group protected compound **10** may influence the transporter selectivity when compared to **11**. At the same time, the distance of carboxylic acids favors a longer distance (**11**) rather than a shorter one (**8**). A comprehensive summary of structure-uptake relationships is presented in Figure 8.

Replacement of the hydroxyl group at the C-3 position of the 3α-hydroxy-5β-androstane skeleton with any of the 3, 4, and 5 carbon chained terminal carboxylic groups (compounds **6**–**10**) improved the affinity of the compounds. This can be attributed to the interactions of carboxylic acid functional group with residues Lys33 and Arg556, as seen in compounds **6**–**10** when compared to compound **1**. Because of the absence of free carboxylic anion in compound **5**, loss of interaction with Arg556 was seen, which could also be related to low affinity of compound **5**. E3S has sulfate group at the C-3 position and shows hydrogen bond interactions with the Lys33 and Arg556 residues and depicts that the hydrogen bond acceptor and anion groups are important at this position for affinity. At the C-17 position of the androstane ring, replacement of acetyl group with methyl or hydrogen has decreased the affinity as compared to E3S. The amine group substitution in 4 carbon amide compounds (compound **6** versus **8**) displayed interactions with Glu172 and Glu200, while losing interaction with Arg168. In the case of 5 carbon amide compounds (compound **9** versus **11**), the addition of an amine group allowed the interaction with Glu172 and Glu200, while retaining Arg168 interaction. This might also explain the increase in the capacity for compound **11**. Addition of the Boc-group on amine has increased the affinity in 4 carbon amide compounds (compound **7** versus **8**), whereas no major effect in the affinity was observed in 5 carbon amide compounds (compound **10** versus **11**).

**Figure 9:**
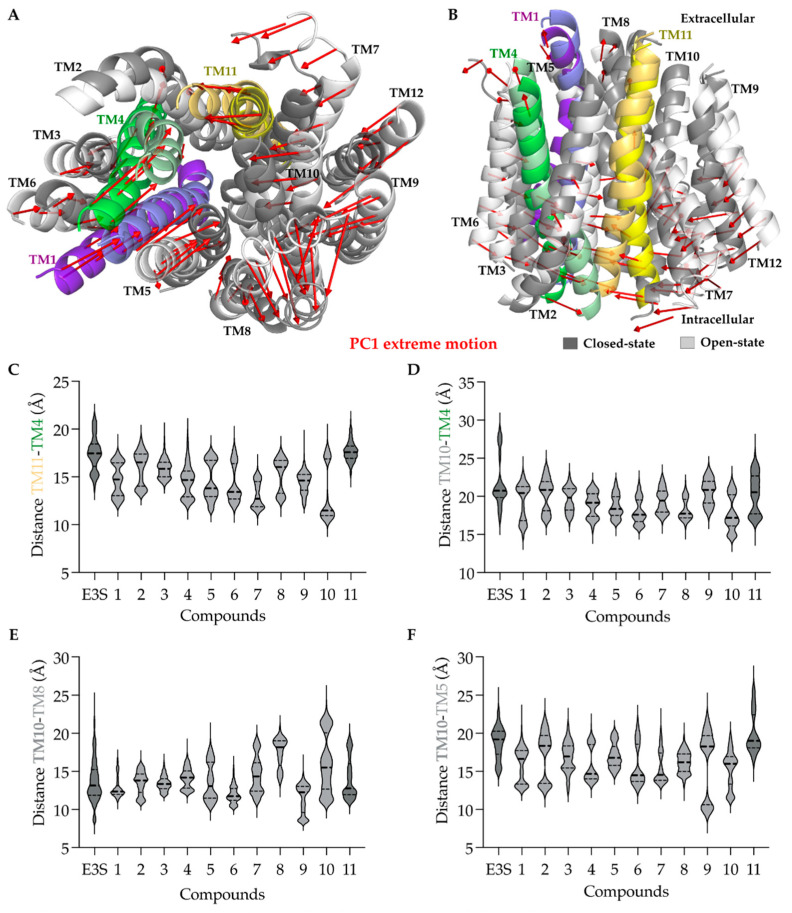
Principal Component Analysis (PCA) of OATP1A2-ligand bound complexes: Principal components analysis revealed two states: open (light gray) and closed (dark gray) from PC1. (**A**) Intracellular view of transmembrane helices displayed closed and open states, and the red arrows show the direction of movement of helices. (**B**) Lateral view of transmembrane helices showing the two states. (C),(D),(E),(F) Centroid distance between two helices measured along the simulations of each protein-ligand complex. (**C**) TM11 residues 544–555 to TM4 residues 173–185. (**D**) TM10 residues 528–538 to TM4 residues 173–185. (**E**) TM10 residues 528–538 to TM8 residues 373–383. (**F**) TM10 residues 528–538 to TM5 residues 192–201.

PCA extreme motions suggested preferred protein conformations for different ligands (Figure 9A,B), especially E3S, and compound **11** often displayed an open conformation, toward the intracellular side, with changes in the TM4, TM5, and TM11 (Figure 9C–F). Residues relevant for the stabilization of the proposed binding mode, such as Arg168, Glu172 (TM4), Glu200 (TM5), and Arg556 (TM11), were present on these transmembrane helices, suggesting the influence of the compound binding on the helical movements and, therefore, in opening-closing transition.

## 4. Materials and Methods

### 4.1. Chemicals

All reagents and solvents used in these studies were commercial and high purity of analytical grade or ultra-gradient LC-MS-grade purchased from MilliporeSigma (St. Louis, MO, USA), J.T. Baker (Deventer, The Netherlands), Merck (Darmstadt, Germany), Riedel-de Haën (Seelze, Germany), or Thermo Fisher Scientific (Waltham, MA, USA), unless otherwise stated. Water was purified using a Milli-Q Gradient system (Millipore, Milford, MA, USA). The synthetic neurosteroids were prepared and analyzed as per our earlier published protocols [32]. The LC-MS confirmed the purity of final compounds as >95%.

### 4.2. Cell Cultures

MCF-7 human breast adenocarcinoma cells (HTB-22; RRID: CVCL_0031) were purchased from the American Type Culture Collection (ATCC, Manassas, VA, USA). The cells were cultured in standard conditions (37 °C, 5% CO_2_) using Dulbecco’s modified Eagle medium (DMEM) supplemented with L-glutamine (2 mM), heat-inactivated fetal bovine serum (10%), penicillin (50 U/mL), and streptomycin (50 μg/mL). Once the cells reached 80% confluence in culture bottles (75 cm^2^), the cells were washed twice with DPBS solution and harvested by trypsinization. All the following experiments were carried out with passage number 9–22.

### 4.3. Cellular Uptake of Neurosteroids

For the cellular uptake experiments, MCF-7 cells were seeded at the density of 1 × 10^5^ cells/well onto 24-well plates a day before the experiments. Cellular uptake of neurosteroids (**1**–**11**) dissolved in DMSO and diluted with HBSS or MES buffer containing 5% 2-hydroxypropyl-β-cyclodextrin (HP-β-CD) was studied by incubating the cells with the pre-warmed compound solutions (250 μL) at the concentrations of 1–200 μM, at pH 7.4 (HBSS buffer), or in parallel at 5.0 (MES buffer), at 37 °C for 30 min (uptake was linear with all compounds up to 30 min). Subsequently, the cells were washed three times with ice-cold buffer and lysed with 250 μL of NaOH (0.1 M) for 60 min. The lysates were diluted with acetonitrile (ACN) with a ratio of 1:3 and centrifuged at 10,000× *g* for 10 min. The samples were analyzed by high-performance liquid chromatography (HPLC) methods described below. The concentrations of each compound were calculated from the standard curve and normalized to protein concentration in each 24-plate that was determined as a mean of three samples by Bio-Rad Protein Assay, based on the Bradford dye-binding method, using BSA as a standard protein and measuring the absorbance (595 nm) by multiplate reader (EnVision, Perkin Elmer, Inc., Waltham, MA, USA).

The competitive uptake of 25 µM neurosteroids (**1**–**11**) in the presence of efflux inhibitors, 50 µM elacridar (P-gp and BCRP), and 50 µM MK-571 (MRPs) at pH 7.4 was carried out as described above, and the concentrations of studied compounds were analyzed with HPLC methods (below), calculated according to the standard curve, and normalized with protein concentration measured to each 24-well plate.

### 4.4. High-Performance Liquid Chromatography (HPLC) Analyses

Concentrations of neurosteroids (**1**–**11**) were determined by HPLC. The apparatus was comprised of an Agilent 1100 binary pump (Agilent Technologies Inc., Wilmington, DE, USA), 1100 micro vacuum degasser, HP 1050 Auto-sampler, HP 1050 variable wavelength detector, operating at 225 nm. The chromatographic separations were achieved on a Supelco Supelcosil LC-Si analytical column (4.6 mm x 250 mm, 5 ml) (Supelco Inc., Bellefonte, PA, USA) by using isocratic elution of water containing 0.1% phosphoric acid and ACN with ratio of 30:70. The retention times of the compounds were ca. 4.3–4.4 min at the flow rate of 1.0 mL/min at room temperature. The lower limit of quantification for the compounds varied from 0.005 to 0.1 μM. These HPLC methods were accurate (100 ± 10% of nominal concentration), precise (RSD% <10%), and selective (no interfering peaks) over the range 0.1–2 μM.

### 4.5. Data Analysis

All data analyses, including Michaelis-Menten and Eadie-Hofstee analyses, were performed using GraphPad Prism v. 5.03 software (GraphPad Software, San Diego, CA, USA). Statistical differences between groups were tested using one-way ANOVA, followed by a two-tailed Tukey’s multiple comparison test and presented as mean ± SD, with statistically significant difference denoted by * *p* < 0.05, ** *p* < 0.01, *** *p* < 0.001.

### 4.6. Molecular Modeling

#### 4.6.1. Homology Model Generation and Protein Preparation

OATP1A2 full-length sequence (UniProt: P46721) was used to generate a homology model. The model was generated based mostly on the bacterial D-galactonate proton symporter (SLC17, PDB ID: 6E9N, resolution 2.92 Å, Chain A), which displays an open conformation towards the cytoplasmic side (see Supporting information, Figure S1). Since the SLC17 template does not cover the region equivalent to the 5th extra-cellular of OATP1A2, an extra template was used, the pancreatic secretory trypsin inhibitor (Kazal type, PDB ID:1TGS, resolution 1.8 Å, Chain I; see Supporting information, Figure S2). Alignment between the sequence and the templates was done in PROMALS3D. Models were generated with Modeller (v.9.23), using standard options, and validated by Ramachandran plot. Around 4% of residues of the model were found in disallowed regions from Ramachandran plot; therefore, the model was subjected to refinement using GalaxyRefine web server. Final refined model was then prepared for docking using Protein Preparation Wizard (implemented in Maestro 2019.4) for adjusting the ionization states of amino acid side-chains (pH 7.0), adding the amino acid charges, which was followed by energy minimization, using the OPLS3e force-field [44].

#### 4.6.2. Binding Site Prediction

SiteMap [45] was used to predict the potential binding regions in the transporter, which predicts possible druggable binding pockets based on the geometrical parameters, such as hydrophobicity, hydrophilicity, exposure to solvent, volume, and enclosure of the protein residues. The predicted sites will be scored using SiteScore and Dscore that provides the druggablility information of the predicted site. SiteScore threshold above 0.8 differentiates between non-druggable and druggable sites [46]. SiteMap was run on the model to identify five potential binding sites using standard grid and more restrictive hydrophobicity definition (see Supporting information, Table S1). The top ranked predicted binding site (Site 1) has SiteScore 1.18, and second top ranked site (Site 2) has SiteScore 1.11. Although Site 1 and Site 2 are similar in their druggability, Site 2 has a larger pocket and is located in a central pore, so this pocket has been selected for docking and MD simulation analysis.

#### 4.6.3. Ligand Preparation and Docking

Ligands were drawn in Maestro, and their 3D conformations were generated using LigPrep [47]. Briefly, LigPrep added hydrogen atoms and adjusted the charges, ionization states were generated using Epik at pH 7.0 ± 1.0 [48]. Three-dimensional configurations of the compounds were generated using OPLS3e force-field, and a conformational search was done using MacroModel conformational sampling methods using default settings [49]. Prepared ligands were docked in the predicted binding site of OATP1A2. The binding site was defined based on the top ranked predicted pocket using SiteMap [45]. Docking was performed using Glide, with the Standard Precision (SP) mode, within a box of 10 Å around the center of the defined binding pockets, and all other options were used as default. Poses were visually inspected to select based on the interactions with known residues from literature and alignment between different ligands. Selected poses were submitted to molecular dynamic simulations to inspect the stability of their binding within the predicted binding site.

#### 4.6.4. Molecular Dynamics (MD) Simulations

Desmond [50] engine was used for the molecular dynamic simulations with the OPLS3e force-field. Systems were prepared using TIP3P solvation model and DMPC (dimyristoylphosphatidylcholine) membrane pre-equilibrated at temperature 300 K and Na^+^ as counterions. Systems were generated as an orthorhombic box, with a buffer distance of 13 Å, using PBC conditions. The membrane was placed perpendicular to the central pore of protein according to the conformation of the helices. Prepared systems were then simulated for at least 500 ns with 3 replicas (with different seeds for the velocities) using NPγT ensemble at 300 K. Simulations were performed using a Nose-Hoover chain thermostat, and the pressure was maintained at 1.01 bar with Martyna-Tobias-Klein barostat. Default relaxation protocol was used with the RESPA integrator with a time step of 2 fs for bonded atom pairs, and non-bonded interactions treatment was separated between near (2 fs) and far (6 fs) atom pairs. A cut-off radius for short range coulombic interactions was set to 9 Å. Atomic interactions and distances were determined using the Simulation Event Analysis pipeline, as implemented in Maestro 2020.2 (Schrödinger LCC). MD trajectories were visualized and figures produced by PyMol v.2.4 (Schrödinger LCC, New York, NY, USA).

#### 4.6.5. Principal Component Analysis (PCA)

Extreme motions of the protein complexes during the molecular dynamics simulations were analyzed using principal component analysis. All the python scripts used in this study were provided by Schrödinger. Analysis was run considering only the variation of the backbone atoms from the transmembrane domains, which were kept using trj_keep_selection_dl.py script. Entire trajectory was then aligned to frame 0 (initial frame) using trj_align.py script, and trajectories from all the simulations were merged using python script trj_merge.py. The combined trajectory was used to generate .xtc and .pdb files (required for the Gromacs software) using trj_no_virt.py script, followed by our in-house developed script (fix_pdb.py), used to fix the pdb file generated in the previous step.

The generated files were used for the PCA analysis using Gromacs (version 2020.4). To exclude the extreme motions incurred by loops (of intracellular and extracellular regions), only transmembrane helices were considered for the calculations using make_ndx script from Gromacs. Further, principal component analysis was carried out using gmx anaeig, following the covariance matrix generation using the gmx covar command line, with standard options. Extreme motion figures were generated and visualized using mode vectors script from PyMol. Distances between helices were calculated using trj_asl_distance.py script from Schrödinger.

## 5. Conclusions

In conclusion, all newly synthesized neurosteroids had relatively low transport capacity into the MCF-7, but there was a significant variation within the transport efficacies between the compounds. Moreover, most of the compounds used two distinct transport mechanism, one of which was with lower capacity (V_max_ values from 3.0 to 26.3 pmol/mg/min) and higher affinity (K_m_ values from 3.0 to 76.4 µM), and the other with higher capacity (V_max_ values from 8.2 and 150.4 pmol/mg/min) and lower affinity (K_m_ values from 25.2 to 959.6 µM). These two mechanisms can be explained either by the affinities of the steroidal compounds to two distinct binding sites in the same OATP (different transport efficiencies) or by the affinities towards two different OATP subtypes (transport efficiencies close to each other), which needs to be studied more thoroughly in the future.

Structurally, a 3α5β-androstane core can be functionalized at both ends of C-3 and C-17 position. At the C-3 position, amidic structures are favored over esters and must possess next to the amide bond a α-amine (protected or unprotected) and a terminal carboxylic acid. Moreover, the length of the residue at the C-3 position should be at least 5 carbons long. Based on the transporter capacities and efficacies, it could be speculated that the functionalization of the acetyl group at the C-17 position might be explored for further exclusive structure-uptake relationship insights.

Finally, computational analysis of neurosteroid compounds suggested that terminal carboxylic group at the C-3 position is relevant for the affinity with OATP1A2, due to potential polar interactions with Lys33 and Arg556 residues.

## Figures and Tables

**Table 1:** Michaelis-Menten Kinetic Parameters Calculated from Eadie-Hofstee Plot Analysis for Transporter-Mediated Cellular Uptake of Compounds **1**–**11**.

	Transport Mechanism Type 1			Transport Mechanism Type 2		
	V_max_ (pmol/mg/min)	K_m_ (µM)	V_max_/K_m_	V_max_ (pmol/mg/min)	K_m_ (µM)	V_max_/K_m_
**1**	26.3 ± 4.2	55.4	0.47	-	-	-
**2**	*n.d. ^a^*	*n.d. ^a^*	*n.d. ^a^*			
**3**	3.0 ± 0.4	7.7	0.39	8.2 ± 0.1	25.2	0.33
**4**	13.9 ± 6.7	232.8	0.059			
**5**	5.3 ± 0.4	76.4	0.07	34.7 ± 7.7	959.6	0.04
**6**	3.9 ± 0.5	13.8	0.28	10.2 ± 0.1	91.26	0.11
**7**	3.0 ± 0.1	3.0	1.0	23.0 ± 0.5	127.4	0.18
**8**	8.0 ± 2.1	10.9	0.73	28.2 ± 0.1	42.8	0.66
**9**	5.5 ± 0.6	9.2	0.60	26.5 ± 3.0	67.8	0.39
**10**	15.4 ± 6.5	23.0	0.67	150.4 ± 9.9	281.6	0.53
**11**	17.2 ± 2.1	24.2	0.71	-	-	-

^a^ n.d. = not determined due to the unsaturation of the transporter.

## Data Availability

Trajectory of molecular dynamics simulations, their representative frames and the initial homology model (in format PDB) are available in the repository Zenodo (doi 10.5281/zenodo.5211147 and 10.5281/zenodo.5255882).

## Supplementary Materials

The following are available online. In particular, alignments, between: OATP1A2 and template structures galactonate symporter PDB ID: 6E9N (Figure S1); OATP1A2 and template structure pancreatic secretory inhibitor kazal type PDB ID: 1TGS for the 5th extracellular loop region (Figure S2). Characterization of the potential predicted binding sites (Table S1); Conserved residues and polymorphic studies (Figure S3); Stable binding mode of compounds 2–5 (Figure S4); Residues having hydrogen bond interactions and water interactions (Figure S5); Root Mean Squared Deviation (RMSD) of protein and individual compounds (Figures S6–S8); and 2D projections of the Principal Component Analysis (PCA) of OATP1A2-ligand bound complexes (Figure S9) are shown.

## Sample Availability

Samples of the compounds are not available from the authors.
