# Supplementary material for: Neurosteroids: Structure-Uptake Relationships and Computational Modeling of Organic Anion Transporting Polypeptides (OATP)1A2
Source: Molecules. 2021 Sep 17;26(18):5662. doi: 10.3390/molecules26185662 (PMC8472597; doi:10.3390/molecules26185662)
Supplement: Supplementary file 1 [file molecules-26-05662-s001.zip › Adla_et_al_Neurosteroids_Supporting info_V2.pdf]

Supporting information

Neurosteroids: Structure-Uptake Relationships and Computational Modeling of Organic Anion Transporting Polypeptides (OATP)1A2

Santosh Kumar Adla [1,2,*,†], Arun Kumar Tonduru [1,†], Thales Kronenberger [1,3], Eva Kudova [2], Antti Poso [1,3] and Kristiina M. Huttunen [1]

[1] School of Pharmacy, Faculty of Health Sciences, University of Eastern Finland, P.O. Box 1627, FI-70211 Kuopio, Finland; arun.tonduru@uef.f (A.K.T.); kronenberger7@gmail.com (T.K.); antti.poso@uef.fi (A.P.); kristiina.huttunen@uef.fi (K.M.H.)
[2] Institute of Organic Chemistry and Biochemistry (IOCB), Czech Academy of Sciences, Flemingovo Namesti 542/2, 160 00 Prague, Czech Republic; eva.kudova@uochb.cas.cz
[3] Department of Medical Oncology and Pneumology, Internal Medicine VIII, University Hospital of Tübingen, Otfried-Müller-Strasse 14, 72076 Tübingen, Germany
* Correspondence: santosh.adla@uef.fi
† These authors contributed equally to this work.

List of Figures:

Figure S1 Alignment between OATP1A2 and template structure galactonate symporter PDB ID: 6E9N
Figure S2 Alignment between OATP1A2 and template structure pancreatic secretory inhibitor kazal type PDB ID: 1TGS for the 5th extracellular loop region
Figure S3 Conserved residues and polymorphic studies
Figure S4 Stable binding mode of compound 2-5
Figure S5 Residues having hydrogen bond interactions and water interactions
Figure S6 Root Mean Squared Deviation (RMSD) of protein and E3S, compounds **1** - **3**
Figure S7 Root Mean Squared Deviation (RMSD) of protein and compounds **4** - **7**
Figure S8 Root Mean Squared Deviation (RMSD) of protein and compounds **8** – **11**
Figure S9 Principal Component Analysis (PCA) of OATP1A2-ligand bound complexes

List of Table:

Table S1 Prediction of the potential binding sites in the OATP1A2 and its druggability as depicted by the Drug and Site scores (where >0.8 is suggested to be druggable) as well as metrics: exposure to the solvent, pocket volume and ratio between hydrogen bond acceptors and donor

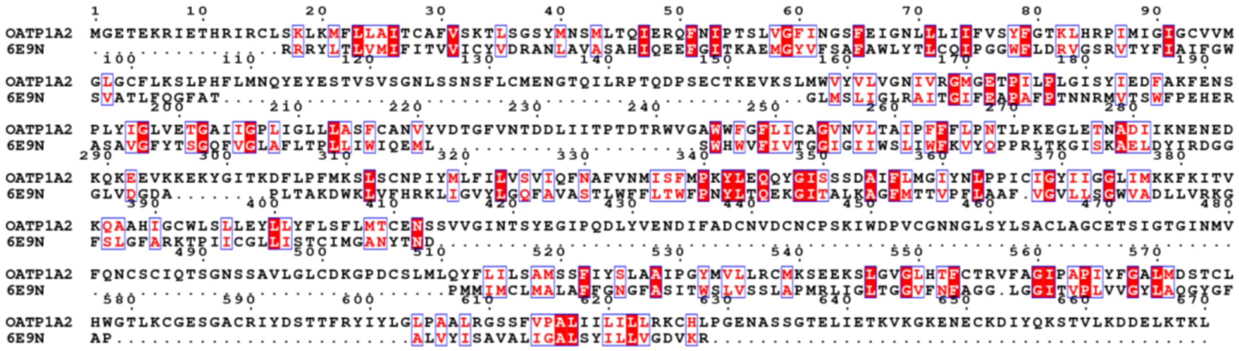

Figure S1. Alignment between OATP1A2 and template structure galactonate symporter PDB ID: 6E9N

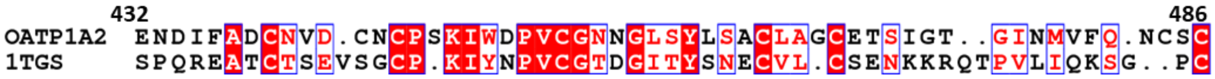

Figure S2. Alignment between OATP1A2 and template structure pancreatic secretory inhibitor kazal type PDB ID: 1TGS for the 5th extracellular loop region

Site	SiteScore	Dscore	Volume (Å)	exposure	hydrophobic	Hydrophilic	Don/acc
Site 1	1.18	1.22	97.06	0.28	4.50	0.45	4.82
**Site 2**	**1.11**	**1.16**	**1264.64**	**0.53**	**1.47**	**0.75**	**0.87**
Site 3	0.98	0.96	590.30	0.64	0.22	1.16	0.93
Site 4	0.97	0.92	381.41	0.57	0.17	1.25	1.05
Site 5	0.77	0.74	185.90	0.68	0.18	1.06	1.04

Table S1. Prediction of the potential binding sites in the OATP1A2 and its druggability as depicted by the Drug and Site scores (where >0.8 is suggested to be druggable) as well as metrics: exposure to the solvent, pocket volume and ratio between hydrogen bond acceptors and donor

A)

33

OATP1A2	------ET--	---HRIR--C	----LSKLKM	FLLAITCAFV	SKTL-SGSYM
OATP1B1	------EN--	---KKTR--Y	----CNGLKM	FLAALSLSFI	AKTL-GAIIM
OATP1B3	------EK--	---KKTR--R	----CNGFKM	FLAALSFSYI	AKAL-GGIIM
OATP1C1	------SE--	---EKQP--C	----CGELKV	FLCALSFVYF	AKAL-AEGYL
OATP3A1	------KK--	---KKKKVSC	----FSNIKI	FLVSECALML	AQGT-VGAYL
OATP2A1	------SRAG	---RCAR-SV	----FGNIKV	FVLCQGLLQL	CQLL-YSAYF
OATP2B1	------GKAS	PDPQDVRPSV	----FHNIKL	FVLCHSLLQL	AQLM-ISGYL
OATP4A1	-VSAGQSVAC	GWWAFAP-PC	LQ-VLNTPKG	ILFFLCAAAF	LQGMTVNGFI
OATP5A1	DLSKTFSVSS	ALAMLQERRC	LYVVLTDSRC	FLVCMCFLTF	IQALMVSGYL
OATP4C1	E-----EGSY	GWRNFHP-QC	LQ-RCNTPGG	FLLHYCLLAV	TQGIVVNGLV
OATP6A1	EVDDSLEQPC	GLGCLVS-TC	CE-CCNNIRC	FMIFYCILLI	CQGVVF-GLI

B)

556

OATP1A2	FIYSLAAIPG	YMVLLRCMKS	EEKSLGVGLH	TFCTRVFAGI	PAPIYFGALM
OATP1B1	FFSALGGTSH	VMLIVKIVQP	ELKSLALGFH	SMVIRALGGI	LAPIYFGALI
OATP1B3	LFSATGGTTF	ILLTVKIVQP	ELKALAMGFQ	SMVIRTLGGI	LAPIYFGALI
OATP1C1	YTLSLGGIPG	YILLLRCIKP	QLKSFALGIY	TLAIRVLAGI	PAPVYFGVLI
OATP3A1	LIGAMAQTPS	VIILIRTVSP	ELKSYALGVL	FLLLRLLGFI	PPPLIFGAGI
OATP2A1	LIACISHNPL	YMMVLRVVNQ	EEKSFAIGVQ	FLLMRLLAWL	PSPALYGLTI
OATP2B1	ALACLTHTPS	FMLILRGVKK	EDKTLAVGIQ	FMFLRILAWM	PSPVIHGSAI
OATP4A1	FFTFLSSIPA	LTATLRCVRD	PQRSFALGIQ	WIVVRILGGI	PGPIAFGWVI
OATP5A1	FITACAQPSA	IIVTLRSVED	EERPFALGMQ	FVLLRTLAYI	PTPIYFGAVI
OATP4C1	IFTFMAGTPI	TVSILRCVNH	RQRSLALGIQ	FMVLRLLGTI	PGPIIFGFTI
OATP6A1	IFSGFSGVPI	VLAMTRVVPD	KLRSLALGVS	YVILRIFGTI	PGPSIFKMSG

C)

168 172

OATP1A2	-ENGTQILRP	TQD--PSECT	KEVKSLMWVY	VLVG-NIVRG	MGETPILPLG
OATP1B1	NQI-LSLNRA	SPEIVGKGCL	KESGSYMWIY	VFMG-NMLRG	IGETPIVPLG
OATP1B3	NQT-LSFNGT	SPEIVEKDCV	KESGSHMWIY	VFMG-NMLRG	IGETPIVPLG
OATP1C1	SQLPVSVMEK	SKSKISNECE	VDTSSSMWIY	VFLG-NLLRG	IGETPIQPLG
OATP3A1	NGSGGD-EGP	DPDLI---CR	NRTATNMMYL	LLIGAQVLLG	IGATPVQPLG
OATP2A1	-KHWQDLPPS	KCHSTTQNPQ	KETS--SMWG	LMVVAQLLAG	IGTVPIQPFG
OATP2B1	-P-TTSAPAS	APSNGNCSSY	TETQHLSVVG	IMFVAQTLLG	VGGVPIQPFG
OATP4A1	--------PA	NPGAVCADST	SGLSR--YQL	VFMLGQFLHG	VGATPLYTLG
OATP5A1	--------TL	EPPA-CPKDS	GGNNHWVYVA	LFICAQILIG	MGSTPIYTLG
OATP4C1	--------TT	RNSTSCTSST	SSL-S-NYLY	VFILGQLLLG	AGGTPLYTLG
OATP6A1	--------EI	KVVSGCQSSG	ISFQS-KYLS	FFILGQTVQG	IAGMPLYILG

Figure S3. Conserved residues and polymorphic studies: **(A)** Shows K33 conserved among OATP1 family, **(B)** Shows R556 conserved among all OATP's, **(C)** Showing polymorphism studied residues R168 and E172 and conserved among the OATP1 family

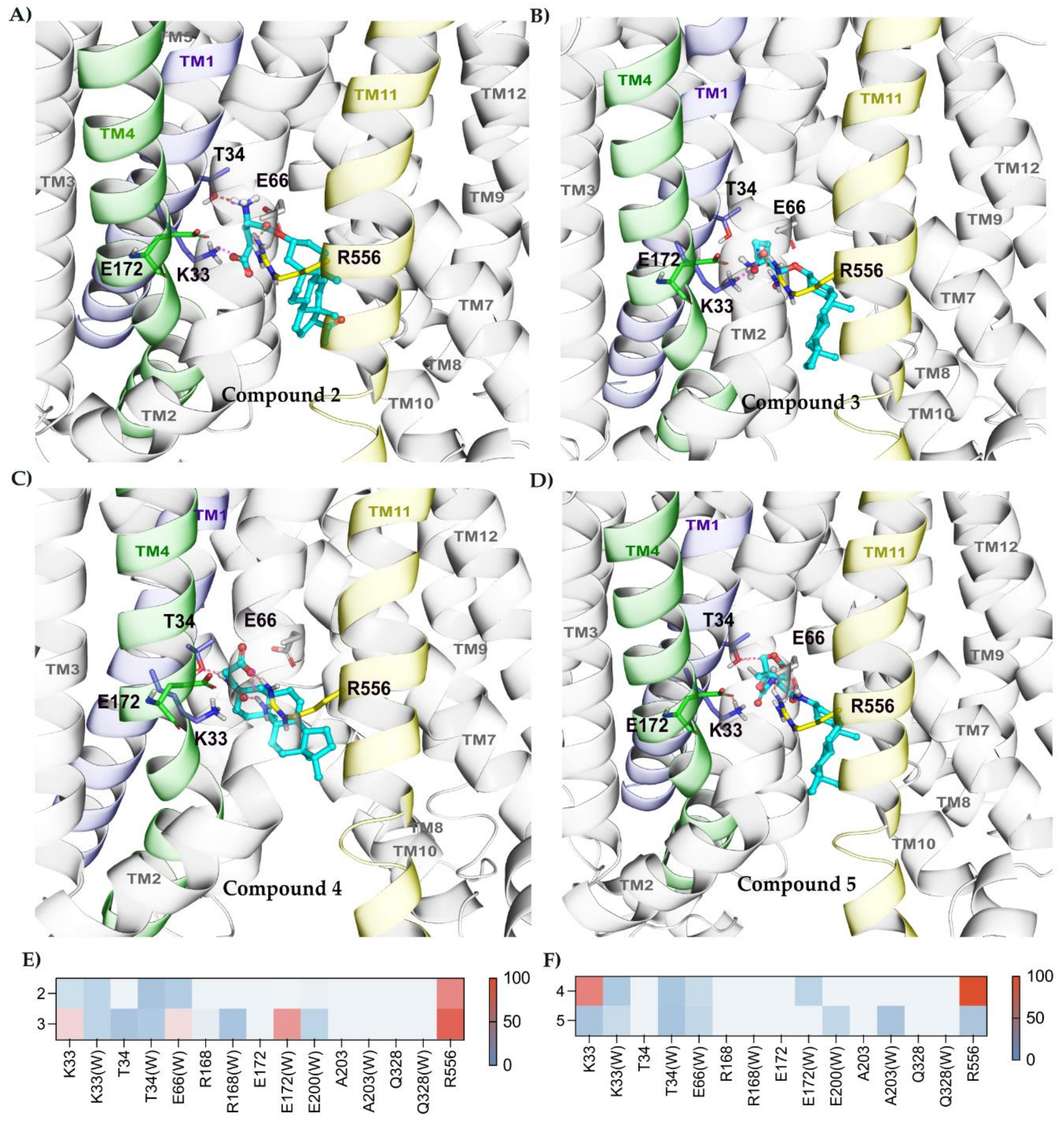

Figure S4. Stable binding mode of compound 2 – 5 relies on polar contacts with K33 and R556. (A) Predicted binding pose of compound 3 in OATP1A2, showing interactions with K33, T34 and R556, **(B)** Predicted binding pose of compound 4 in OATP1A2 showing interactions with K33 and R556, **(C)** Predicted binding pose of compound 5 in OATP1A2 showing interactions with T34 and R556, **(D)** Predicted binding pose of compound 6 showing interactions with T34 and R556, **(E)** and **(F)** Individual residues and hydrogen bond interactions, water interactions (represented as W), for each compound (E - **compounds 2** and **3**, F - **compounds 4** and **5**) are shown as heatmaps, where each interaction is colored according to the frequency.

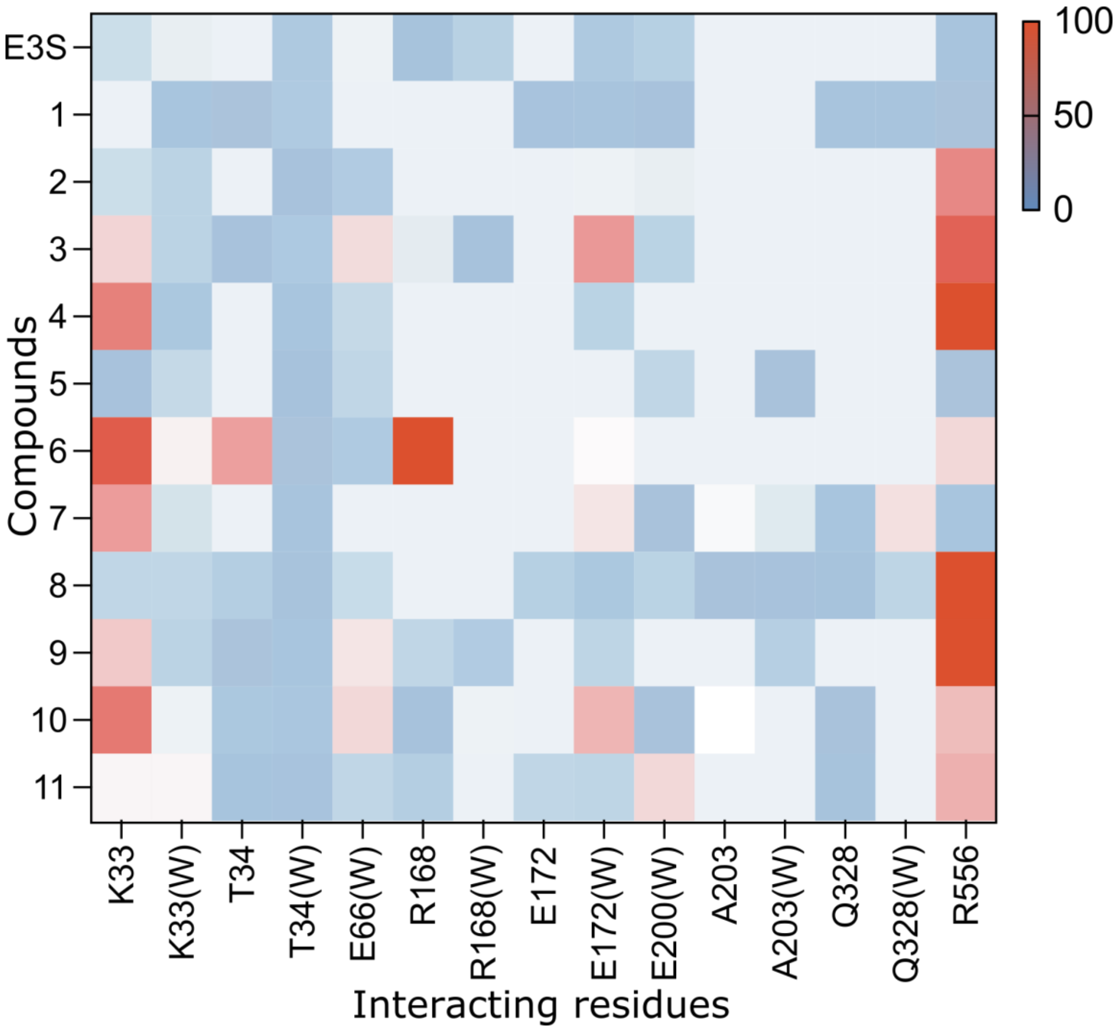

Figure S5. Residues having hydrogen bond interactions and water interactions are shown as heatmaps for all compounds where interaction is colored according to the frequency.

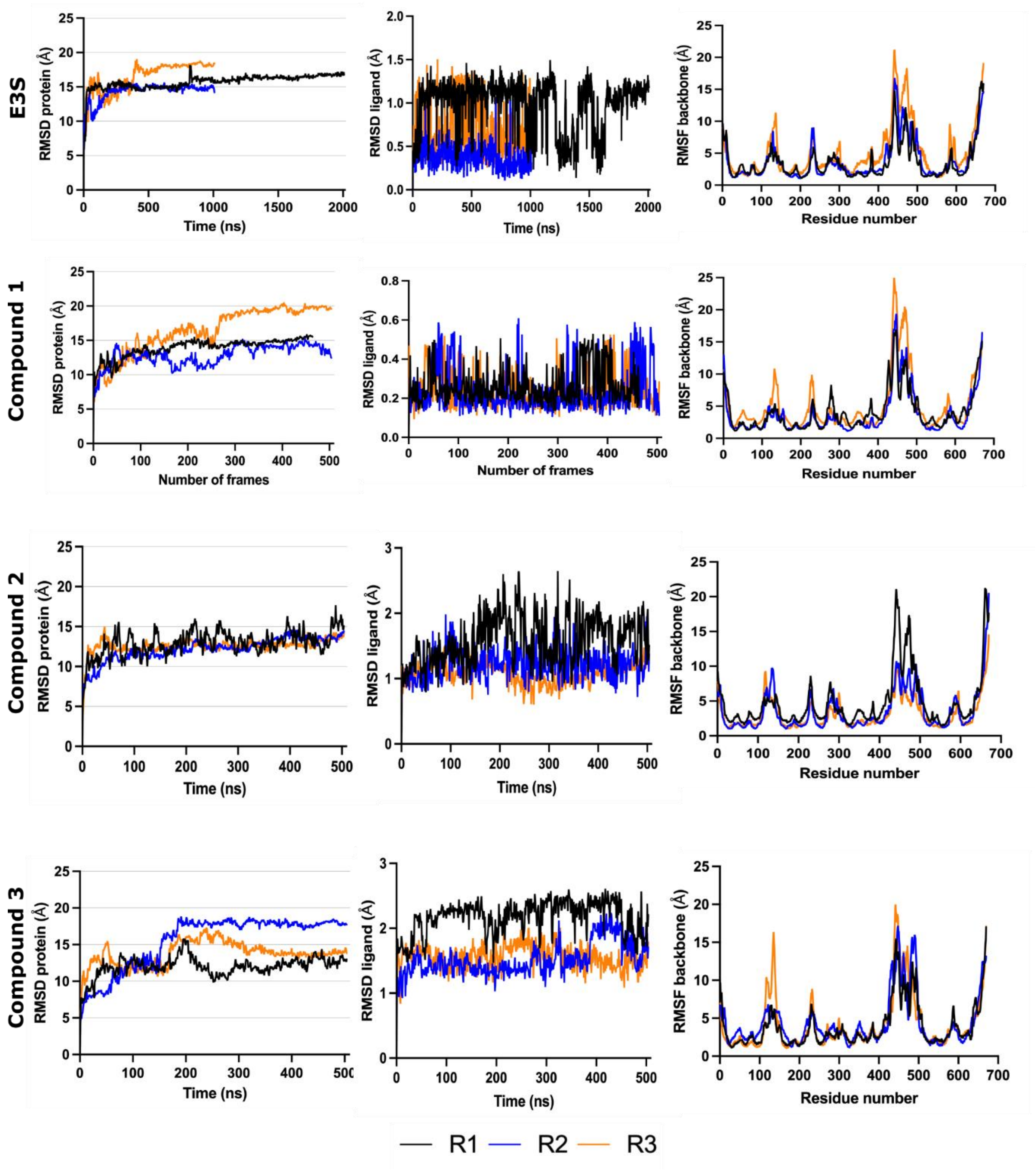

Figure S6. Root Mean Squared Deviation (RMSD) of protein along the analyzed simulation calculated in reference to the initial back bone conformation, **RMSD of ligand** along the analyzed simulation calculated in reference to the initial conformation heavy atoms and **RMSF (Root Mean squared fluctuations) of protein** along the analyzed simulations in reference to the initial conformation of protein backbone for **E3S** and **compounds 1 – 3**. Each replica is represented in different color.

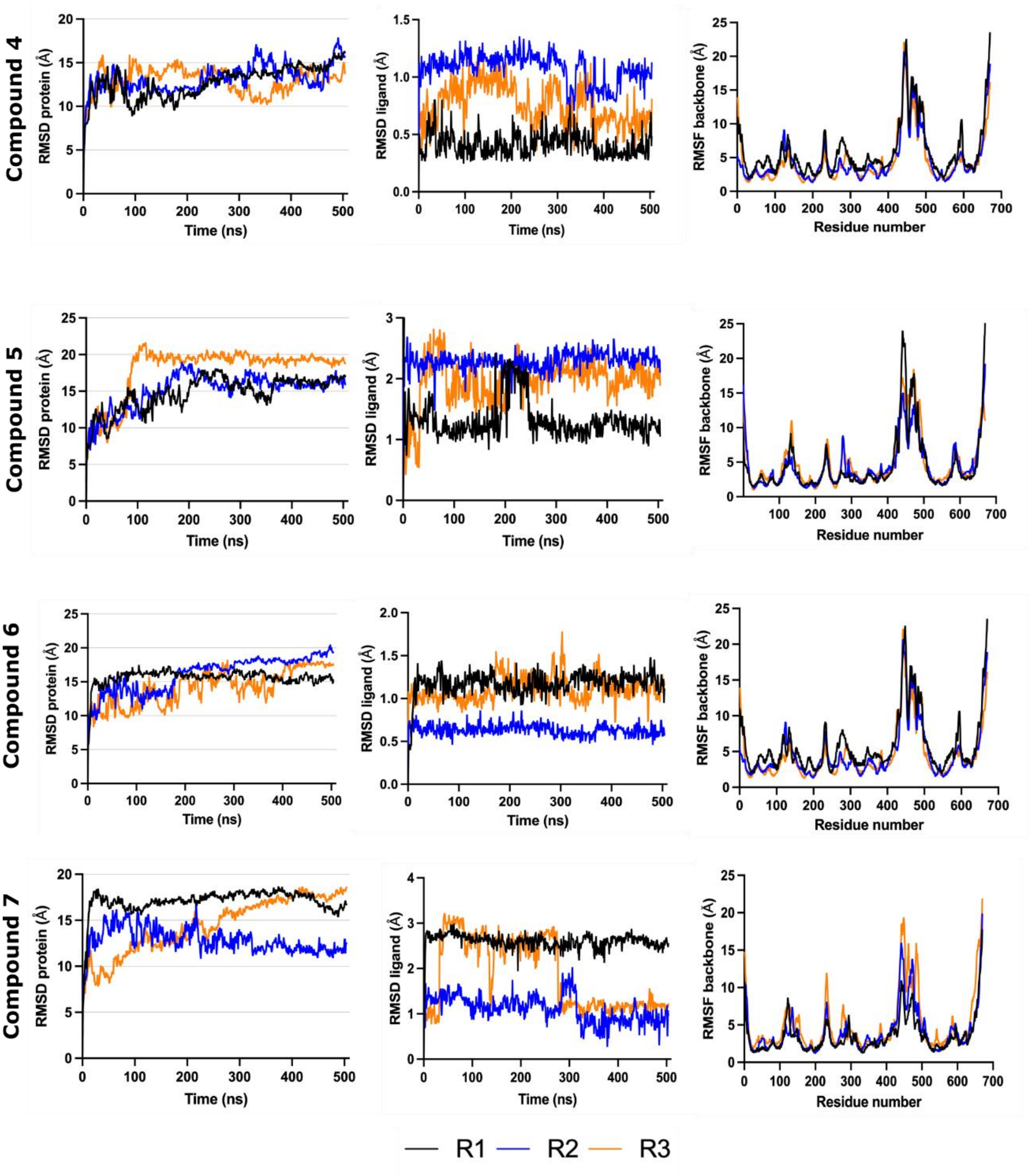

Figure S7. Root Mean Squared Deviation (RMSD) of protein along the analyzed simulation calculated in reference to the initial back bone conformation, **RMSD of ligand** along the analyzed simulation calculated in reference to the initial conformation heavy atoms and **RMSF (Root Mean squared fluctuations) of protein** along the analyzed simulations in reference to the initial conformation of protein backbone for **compounds 4-7**. Each replica is represented in different color.

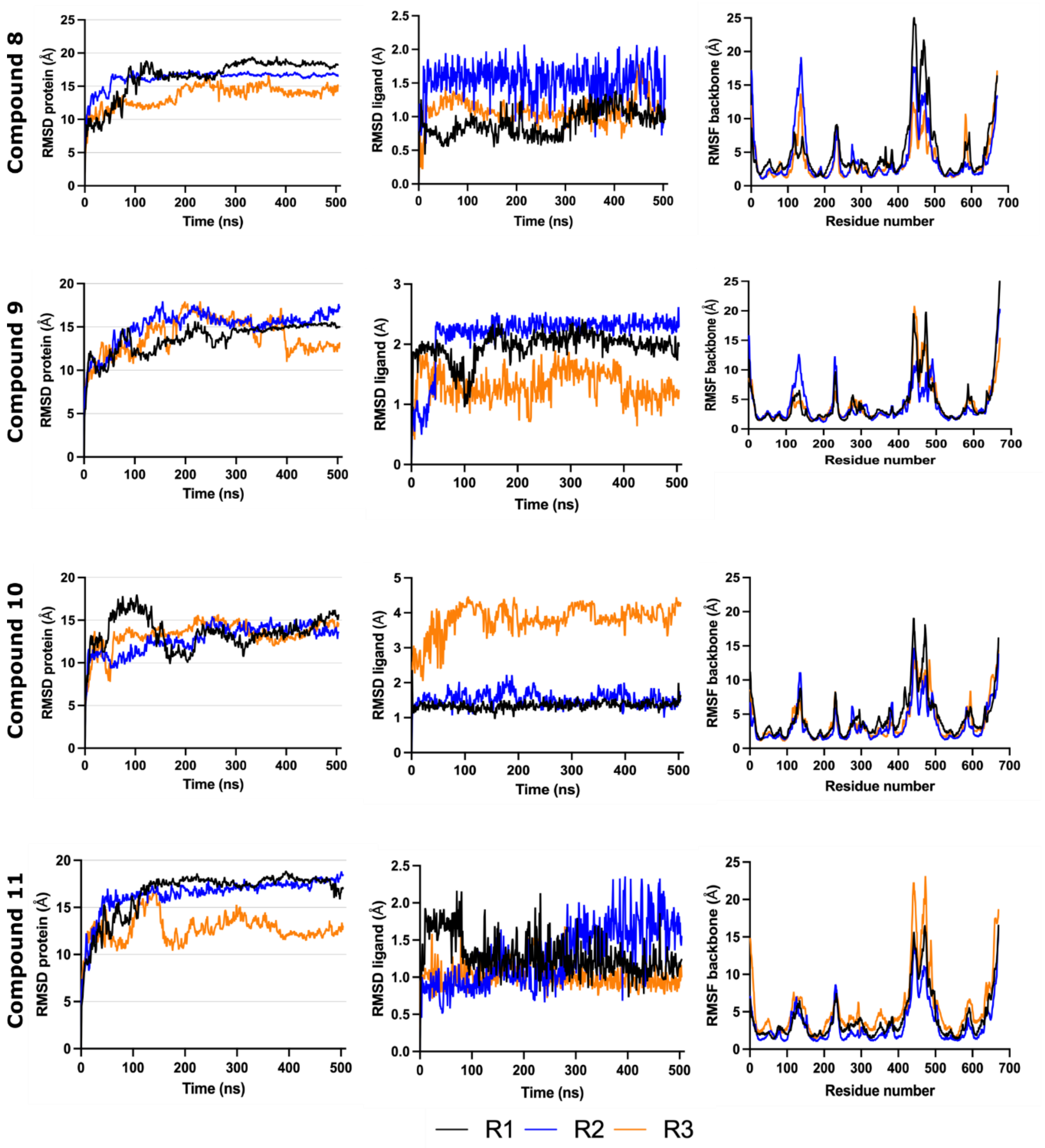

Figure S8. Root Mean Squared Deviation (RMSD) of protein along the analyzed simulation calculated in reference to the initial back bone conformation, **RMSD of ligand** along the analyzed simulation calculated in reference to the initial conformation heavy atoms and **RMSF (Root Mean squared fluctuations) of protein** along the analyzed simulations in reference to the initial conformation of protein backbone for **compounds 8-11**. Each replica is represented in different color.

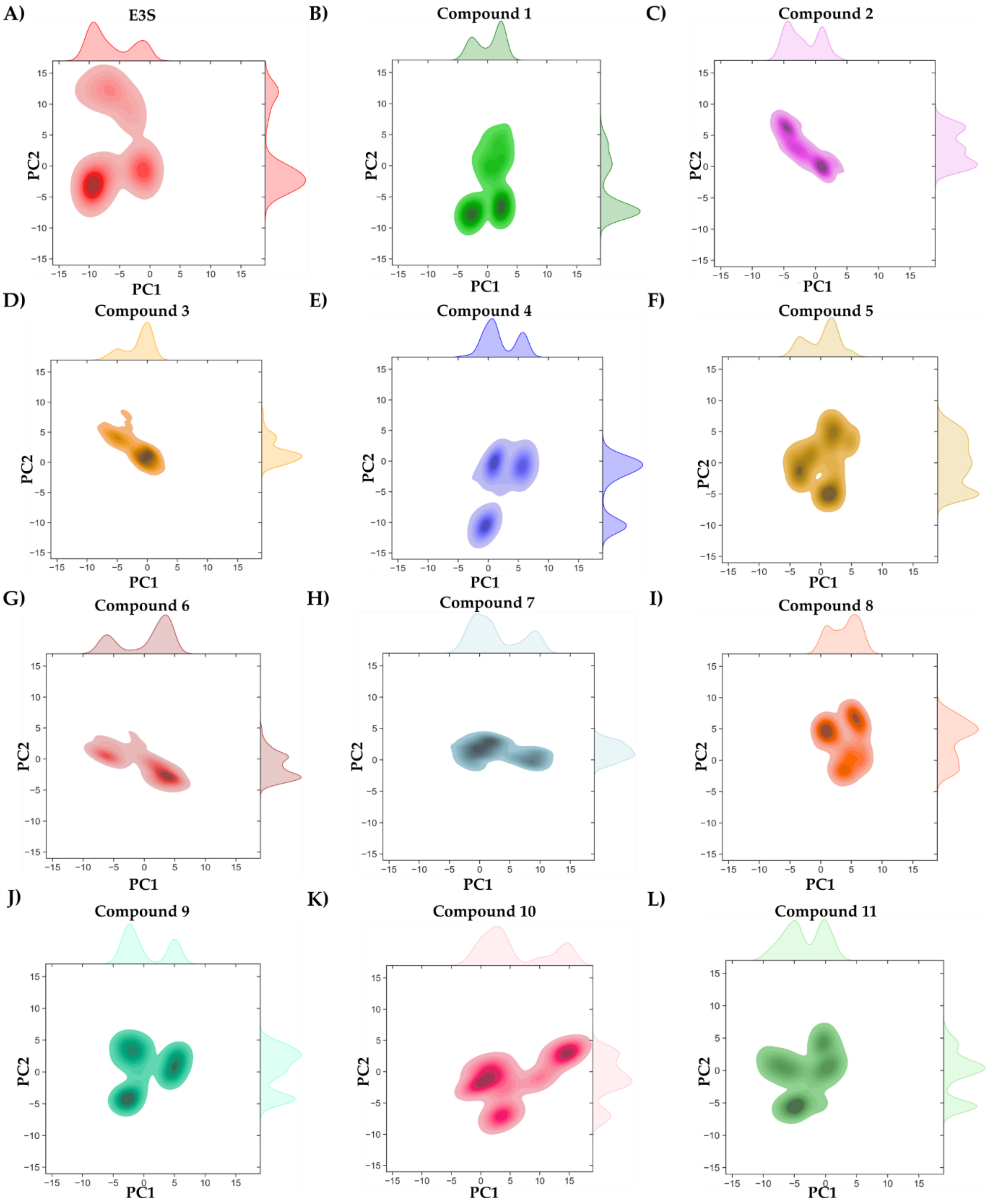

Figure S9. Principal Component Analysis (PCA) of OATP1A2-ligand bound complexes: Principal components plotted PC1 (21.83%) on x-axis vs PC2 (17.81%) on y-axis. A-L). Principal components plotted for all the compounds individually.
