# Supplementary material for: Neurosteroids: Structure-Uptake Relationships and Computational Modeling of Organic Anion Transporting Polypeptides (OATP)1A2
Source: Molecules. 2021 Sep 17;26(18):5662. doi: 10.3390/molecules26185662 (PMC8472597; doi:10.3390/molecules26185662)
Supplement: Supplementary file 1 [file molecules-26-05662-s001.zip › Figure_S1.pdf]

```
         1        10        20        30        40        50        60        70        80        90
OATP1A2  MGETEKRIETHRIRCLSKLKMFLLAITCAFVSKTLSGSYMNSMLTQIERQFNIPTSLVGFINGSFEIGNLLLIIFVSYFGTKLHRPIMIGIGCVVM
6E9N     .................RRYLTLVMIFITVVICYVDRANLAVASAHIQEEFGITKAEMGYVFSAFAWLYTLCQIPGGWFLDRVGSRVTYFIAIFGW
            100       110       120       130       140       150       160       170       180       190
OATP1A2  GLGCFLKSLPHFLMNQYEYESTVSVSGNLSSNSFLCMENGTQILRPTQDPSECTKEVKSLMWVYVLVGNIVRGMGETPILPLGISYIEDFAKFENS
6E9N     SVATLFQGFAT.................................................GLMSLIGLRAITGIFEAPAFPTNNRMVTSWFPEHER
                200       210       220       230       240       250       260       270       280
OATP1A2  PLYIGLVETGAIIGPLIGLLLASFCANVYVDTGFVNTDDLIITPTDTRWVGAWWFGFLICAGVNVLTAIPFFFLPNTLPKEGLETNADIIKNENED
6E9N     ASAVGFYTSGQFVGLAFLTPLLIWIQEML......................SWHWVFIVTGGIGIIWSLIWFKVYQPPRLTKGISKAELDYIRDGG
          290       300       310       320       330       340       350       360       370       380
OATP1A2  KQKEEVKKEKYGITKDFLPFMKSLSCNPIYMLFILVSVIQFNAFVNMISFMPKYLEQQYGISSSDAIFLMGIYNLPPICIGYIIGGLIMKKFKITV
6E9N     GLVDGDA........PLTAKDWKLVFHRKLIGVYLGQFAVASTLWFFLTWFPNYLTQEKGITALKAGFMTTVPFLAAF.VGVLLSGWVADLLVRKG
              390       400       410       420       430       440       450       460       470       480
OATP1A2  KQAAHIGCWLSLLEYLLYFLSFLMTCENSSVVGINTSYEGIPQDLYVENDIFADCNVDCNCPSKIWDPVCGNNGLSYLSACLAGCETSIGTGINMV
6E9N     FSLGFARKTPIICGLLISTCIMGANYTND...................................................................
                  490       500       510       520       530       540       550       560       570
OATP1A2  FQNCSCIQTSGNSSAVLGLCDKGPDCSLMLQYFLILSAMSSFIYSLAAIPGYMVLLRCMKSEEKSLGVGLHTFCTRVFAGIPAPIYFGALMDSTCL
6E9N     ..............................PMMIMCLMALAFFGNGFASITWSLVSSLAPMRLIGLTGGVFNFAGG.LGGITVPLVVGYLAQGYGF
            580       590       600       610       620       630       640       650       660       670
OATP1A2  HWGTLKCGESGACRIYDSTTFRYIYLGLPAALRGSSFVPALIILILLRKCHLPGENASSGTELIETKVKGKENECKDIYQKSTVLKDDELKTKL
6E9N     AP.........................ALVYISAVALIGALSYILLVGDVKR..........................................
```
