# Supplementary material for: Neurosteroids: Structure-Uptake Relationships and Computational Modeling of Organic Anion Transporting Polypeptides (OATP)1A2
Source: Molecules. 2021 Sep 17;26(18):5662. doi: 10.3390/molecules26185662 (PMC8472597; doi:10.3390/molecules26185662)
Supplement: Supplementary file 1 [file molecules-26-05662-s001.zip › Figure_S2.pdf]

```
          1        10        20        30        40        50
          .        .         .         .         .         .
OATP1A2   ENDIFADCNVD.CNCPSKIWDPVCGNNGLSYLSACLAGCETSIGT..GINMVFQ.NCSC
1TGS      SPQREATCTSEVSGCP.KIYNPVCGTDGITYSNECVL.CSENKKRQTPVLIQKSG..PC
```
