# Supplementary material for: Neurosteroids: Structure-Uptake Relationships and Computational Modeling of Organic Anion Transporting Polypeptides (OATP)1A2
Source: Molecules. 2021 Sep 17;26(18):5662. doi: 10.3390/molecules26185662 (PMC8472597; doi:10.3390/molecules26185662)
Supplement: Supplementary file 1 [file molecules-26-05662-s001.zip › Figure_S3.pdf]

A)

```
                                                        33
OATP1A2 ------ET--  ---HRIR--C  ----LSKLKM  FLLAITCAFV  SKTL-SGSYM
OATP1B1 ------EN--  ---KKTR--Y  ----CNGLKM  FLAALSLSFI  AKTL-GAIIM
OATP1B3 ------EK--  ---KKTR--R  ----CNGFKM  FLAALSFSYI  AKAL-GGIIM
OATP1C1 ------SE--  ---EKQP--C  ----CGELKV  FLCALSFVYF  AKAL-AEGYL
OATP3A1 ------KK--  ---KKKKVSC  ----FSNIKI  FLVSECALML  AQGT-VGAYL
OATP2A1 ------SRAG  ---RCAR-SV  ----FGNIKV  FVLCQGLLQL  CQLL-YSAYF
OATP2B1 ------GKAS  PDPQDVRPSV  ----FHNIKL  FVLCHSLLQL  AQLM-ISGYL
OATP4A1 -VSAGQSVAC  GWWAFAP-PC  LQ-VLNTPKG  ILFFLCAAAF  LQGMTVNGFI
OATP5A1 DLSKTFSVSS  ALAMLQERRC  LYVVLTDSRC  FLVCMCFLTF  IQALMVSGYL
OATP4C1 E-----EGSY  GWRNFHP-QC  LQ-RCNTPGG  FLLHYCLLAV  TQGIVVNGLV
OATP6A1 EVDDSLEQPC  GLGCLVS-TC  CE-CCNNIRC  FMIFYCILLI  CQGVVF-GLI
```

B)

```
                                            556
OATP1A2 FIYSLAAIPG  YMVLLRCMKS  EEKSLGVGLH  TFCTRVFAGI  PAPIYFGALM
OATP1B1 FFSALGGTSH  VMLIVKIVQP  ELKSLALGFH  SMVIRALGGI  LAPIYFGALI
OATP1B3 LFSATGGTTF  ILLTVKIVQP  ELKALAMGFQ  SMVIRTLGGI  LAPIYFGALI
OATP1C1 YTLSLGGIPG  YILLLRCIKP  QLKSFALGIY  TLAIRVLAGI  PAPVYFGVLI
OATP3A1 LIGAMAQTPS  VIILIRTVSP  ELKSYALGVL  FLLLRLLGFI  PPPLIFGAGI
OATP2A1 LIACISHNPL  YMMVLRVVNQ  EEKSFAIGVQ  FLLMRLLAWL  PSPALYGLTI
OATP2B1 ALACLTHTPS  FMLILRGVKK  EDKTLAVGIQ  FMFLRILAWM  PSPVIHGSAI
OATP4A1 FFTFLSSIPA  LTATLRCVRD  PQRSFALGIQ  WIVVRILGGI  PGPIAFGWVI
OATP5A1 FITACAQPSA  IIVTLRSVED  EERPFALGMQ  FVLLRTLAYI  PTPIYFGAVI
OATP4C1 IFTFMAGTPI  TVSILRCVNH  RQRSLALGIQ  FMVLRLLGTI  PGPIIFGFTI
OATP6A1 IFSGFSGVPI  VLAMTRVVPD  KLRSLALGVS  YVILRIFGTI  PGPSIFKMSG
```

C)

```
                                                    168   172
OATP1A2 -ENGTQILRP  TQD--PSECT  KEVKSLMWVY  VLVG-NIVRG  MGETPILPLG
OATP1B1 NQI-LSLNRA  SPEIVGKGCL  KESGSYMWIY  VFMG-NMLRG  IGETPIVPLG
OATP1B3 NQT-LSFNGT  SPEIVEKDCV  KESGSHMWIY  VFMG-NMLRG  IGETPIVPLG
OATP1C1 SQLPVSVMEK  SKSKISNECE  VDTSSSMWIY  VFLG-NLLRG  IGETPIQPLG
OATP3A1 NGSGGD-EGP  DPDLI---CR  NRTATNMMYL  LLIGAQVLLG  IGATPVQPLG
OATP2A1 -KHWQDLPPS  KCHSTTQNPQ  KETS--SMWG  LMVVAQLLAG  IGTVPIQPFG
OATP2B1 -P-TTSAPAS  APSNGNCSSY  TETQHLSVVG  IMFVAQTLLG  VGGVPIQPFG
OATP4A1 --------PA  NPGAVCADST  SGLSR--YQL  VFMLGQFLHG  VGATPLYTLG
OATP5A1 --------TL  EPPA-CPKDS  GGNNHWVYVA  LFICAQILIG  MGSTPIYTLG
OATP4C1 --------TT  RNSTSCTSST  SSL-S-NYLY  VFILGQLLLG  AGGTPLYTLG
OATP6A1 --------EI  KVVSGCQSSG  ISFQS-KYLS  FFILGQTVQG  IAGMPLYILG
```
