# Supplementary figures and images for: Neurosteroids: Structure-Uptake Relationships and Computational Modeling of Organic Anion Transporting Polypeptides (OATP)1A2
Source: Molecules. 2021 Sep 17;26(18):5662. doi: 10.3390/molecules26185662 (PMC8472597; doi:10.3390/molecules26185662)

A)

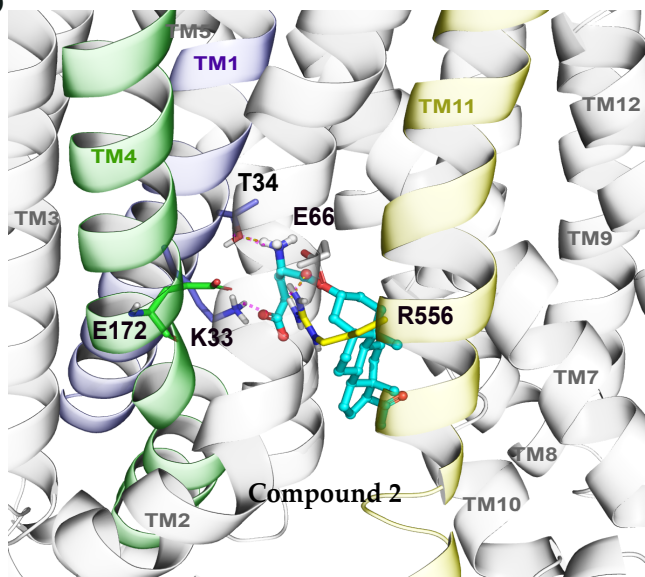

B)

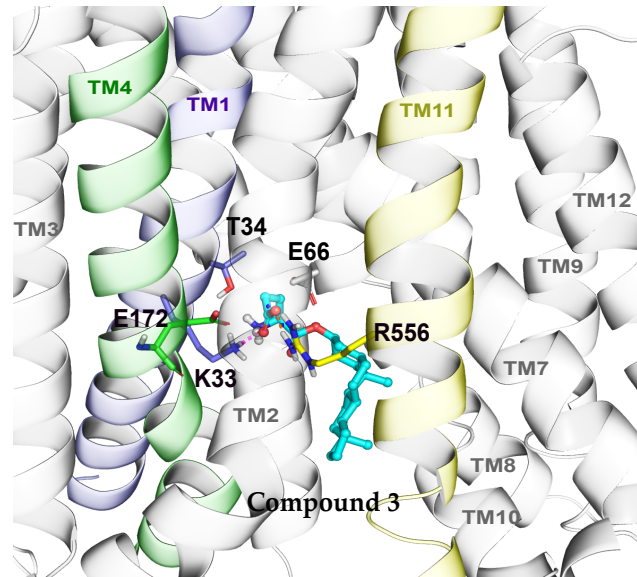

C)

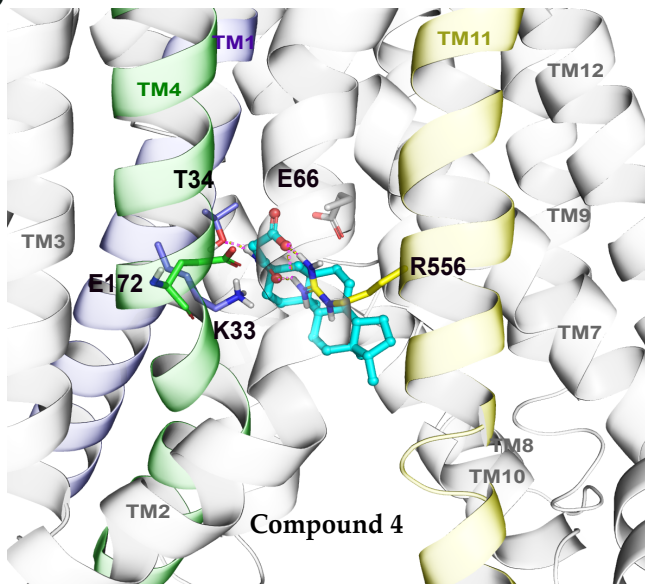

D)

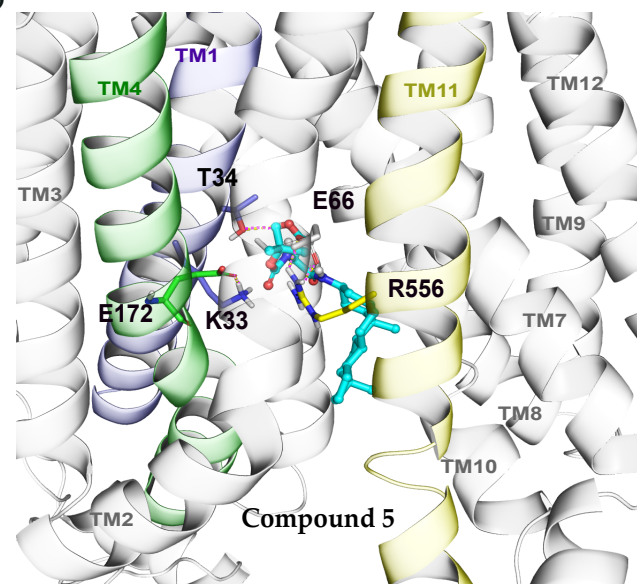

E)

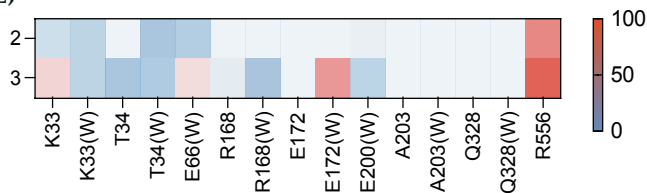

F)

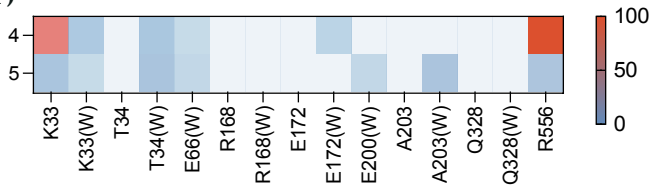

Supplement: Supplementary file 1 [file molecules-26-05662-s001.zip › Figure_S4.pdf]

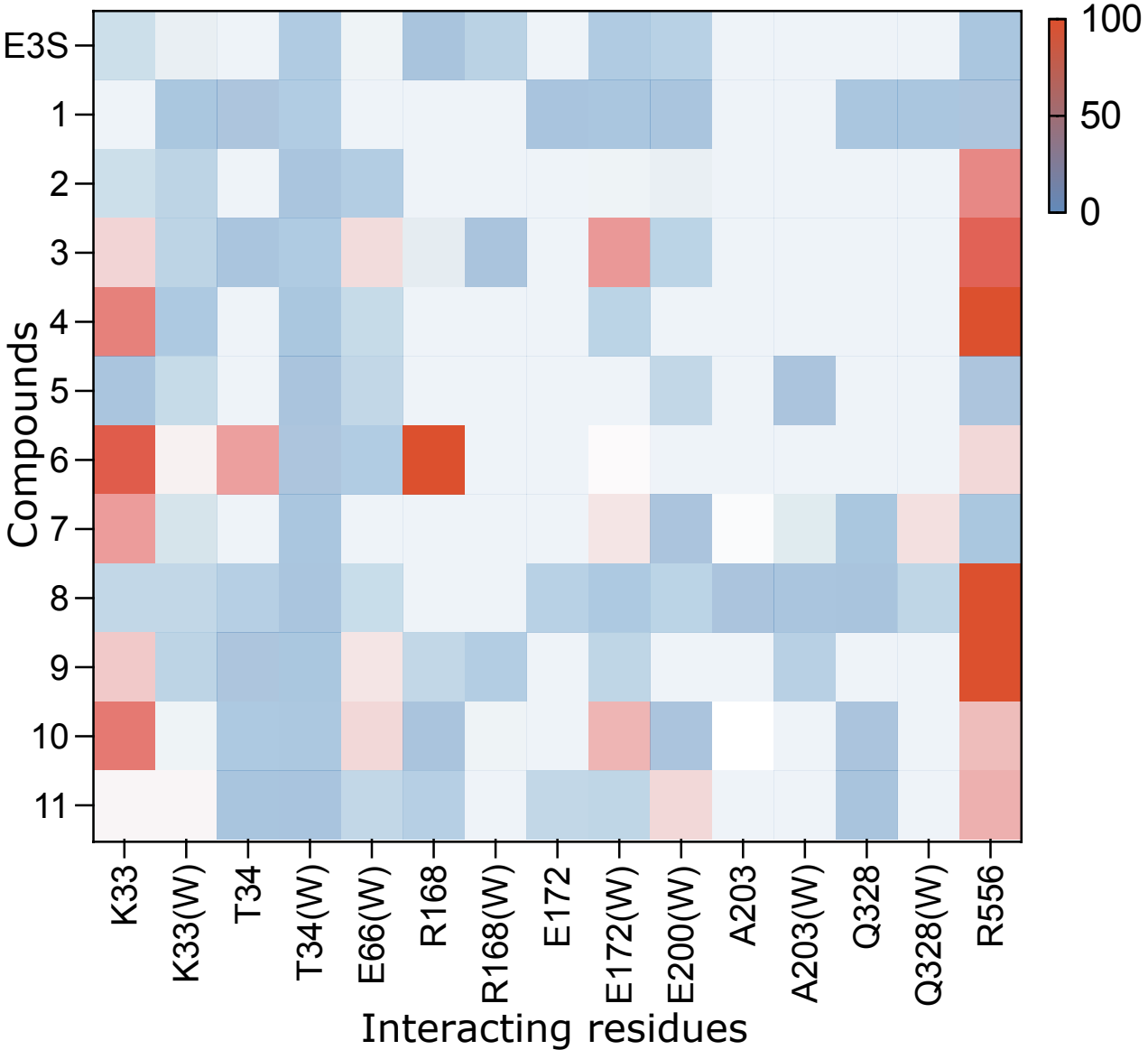

Supplement: Supplementary file 1 [file molecules-26-05662-s001.zip › Figure_S5.pdf]

**E3S**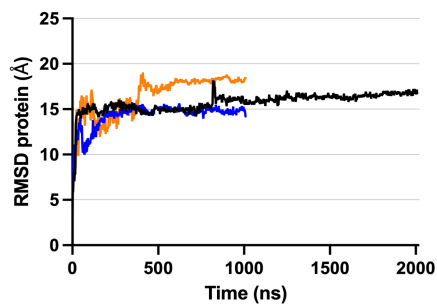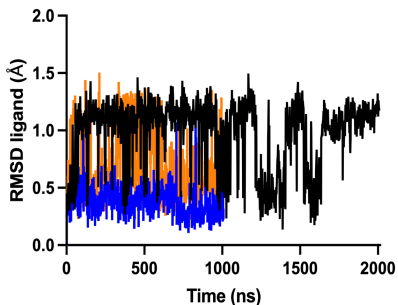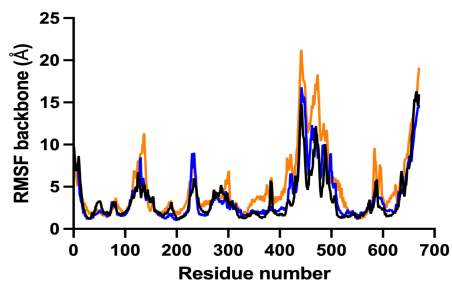**Compound 1**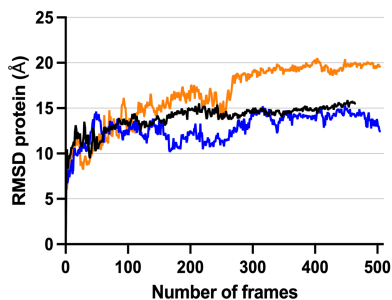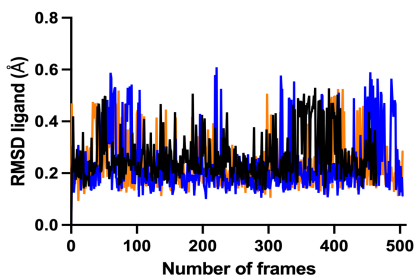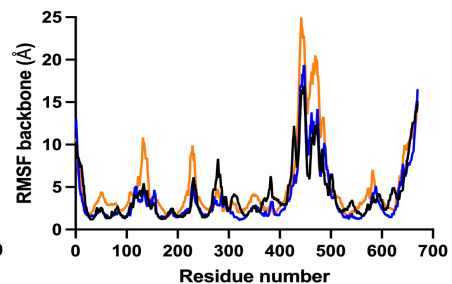**Compound 2**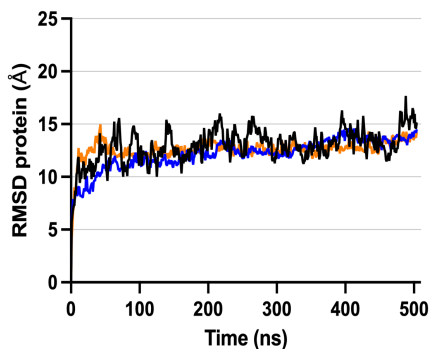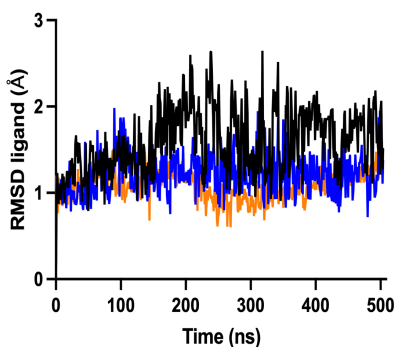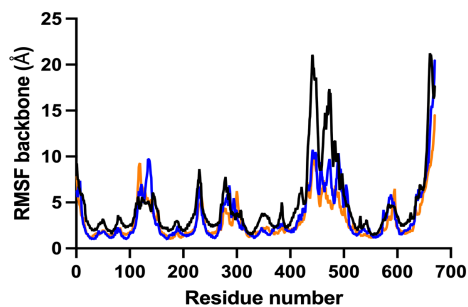**Compound 3**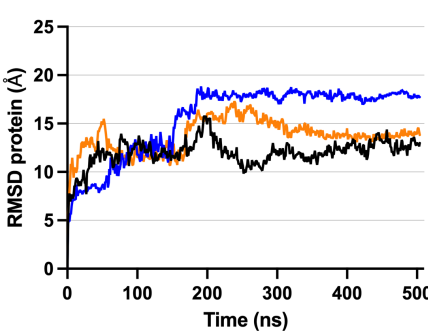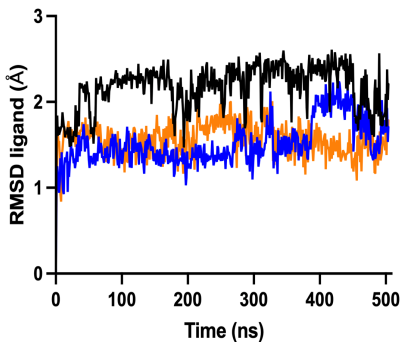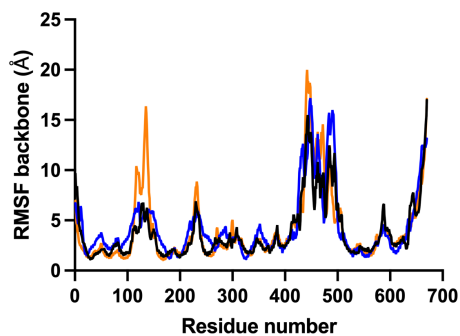

— R1 — R2 — R3

Supplement: Supplementary file 1 [file molecules-26-05662-s001.zip › Figure_S6.pdf]

## Compound 4

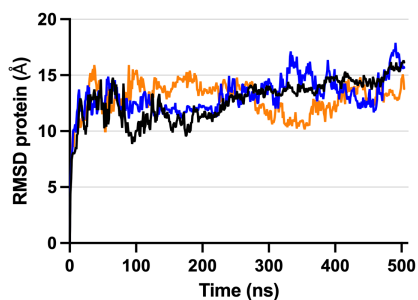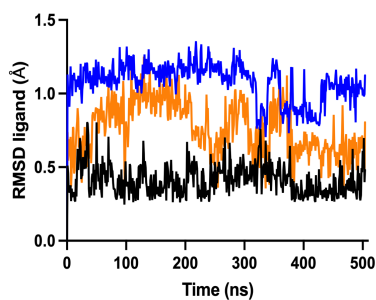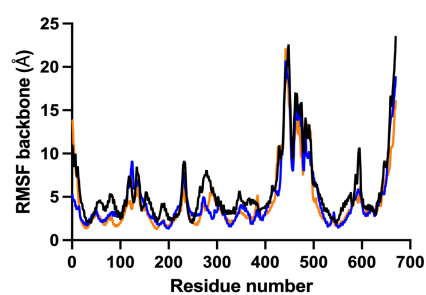

## Compound 5

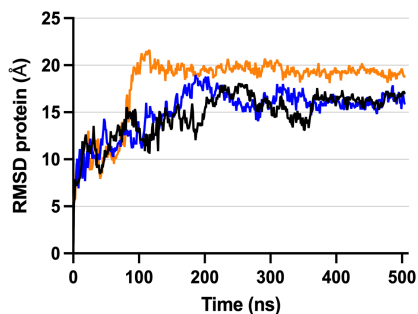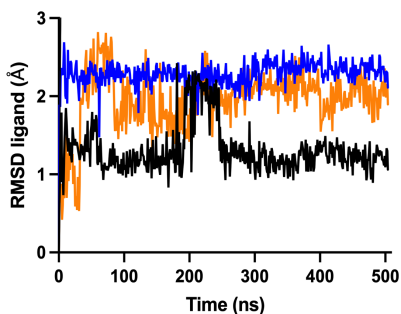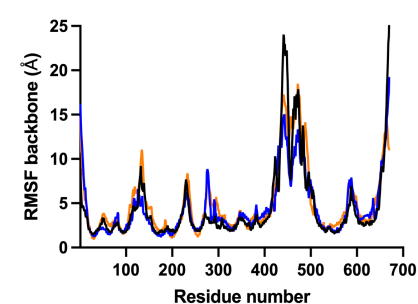

## Compound 6

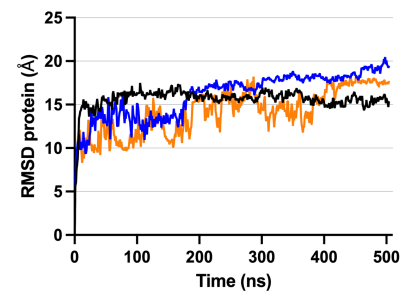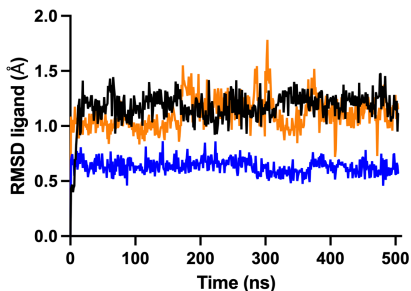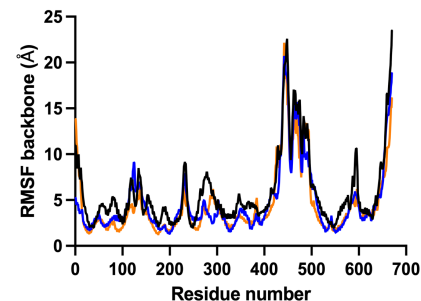

## Compound 7

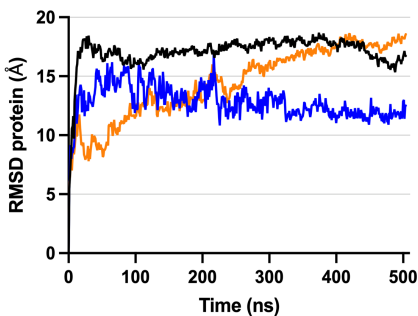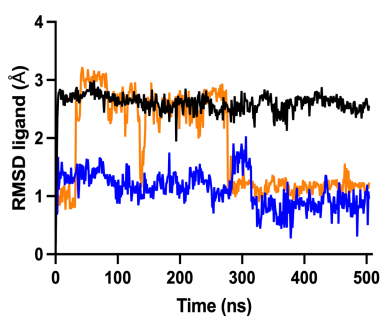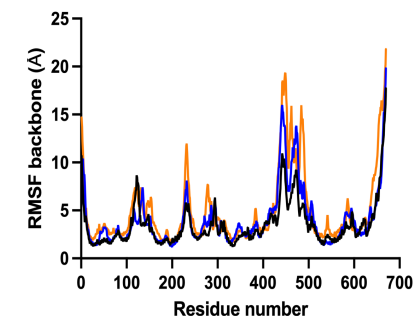

— R1 — R2 — R3

Supplement: Supplementary file 1 [file molecules-26-05662-s001.zip › Figure_S7.pdf]

# Compound 8

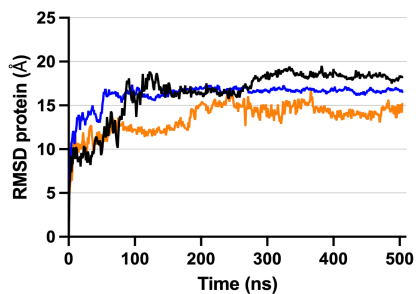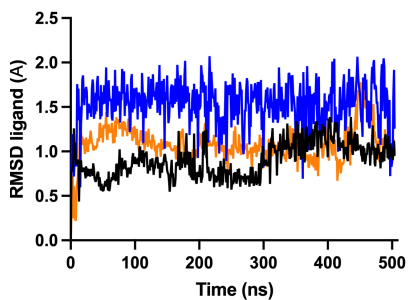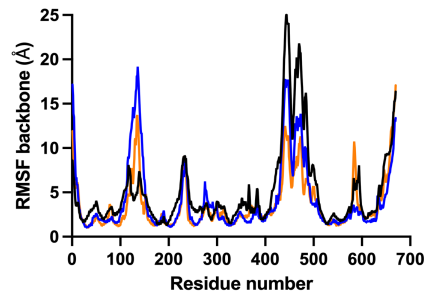

# Compound 9

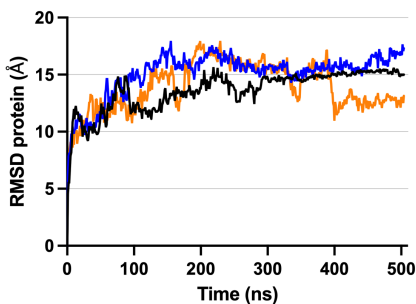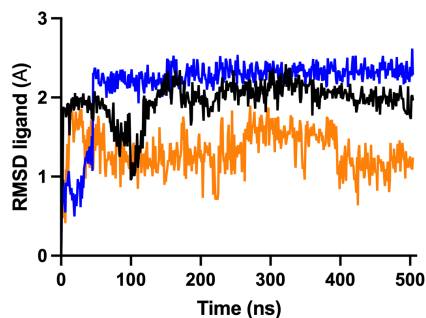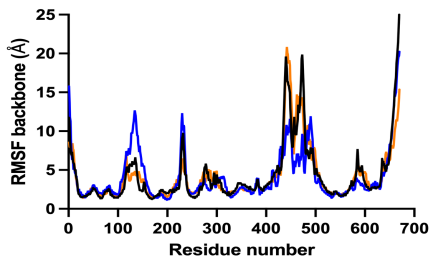

# Compound 10

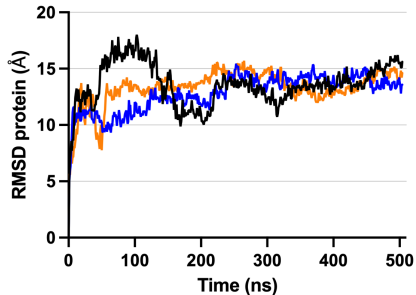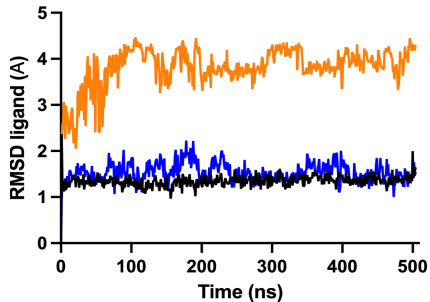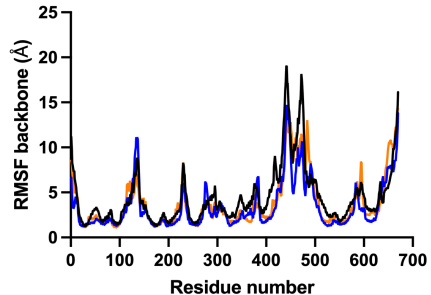

# Compound 11

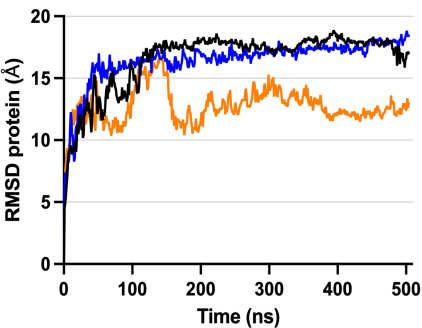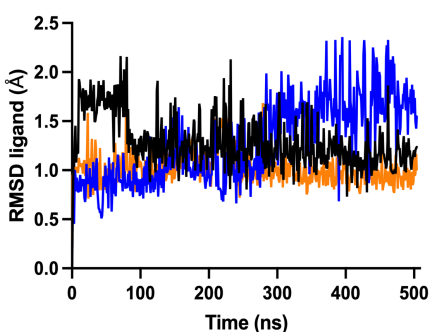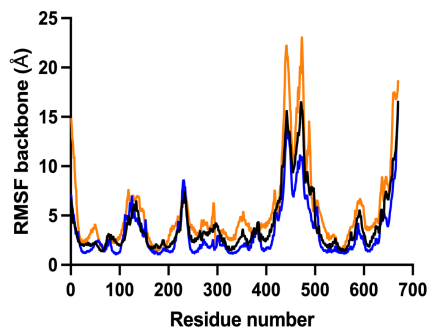

— R1 — R2 — R3

Supplement: Supplementary file 1 [file molecules-26-05662-s001.zip › Figure_S8.pdf]

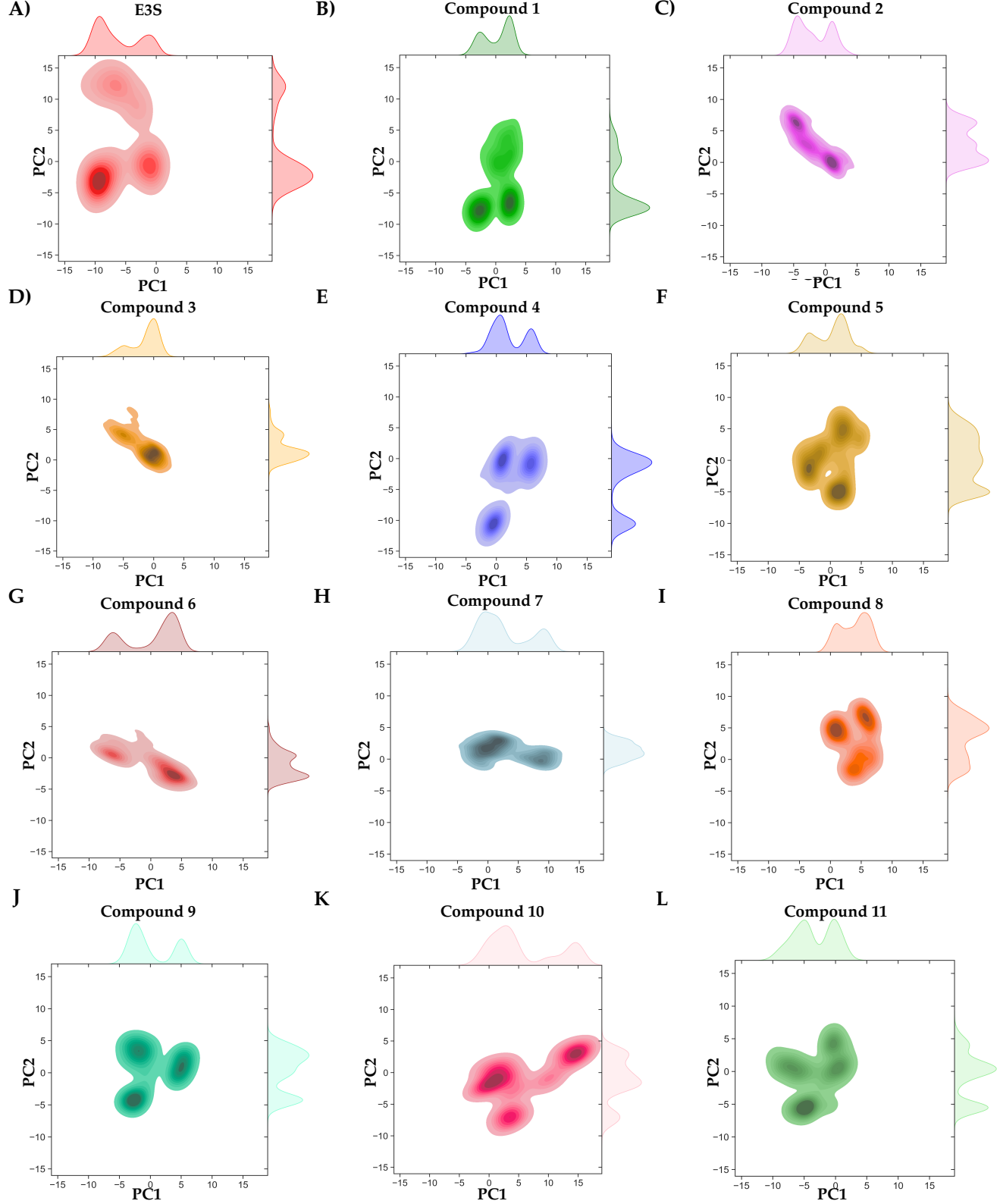

Supplement: Supplementary file 1 [file molecules-26-05662-s001.zip › Figure_S9.pdf]
